# Clinical implications of plasma ctDNA features and dynamics in gastric cancer treated with HER2‐targeted therapies

**DOI:** 10.1002/ctm2.254

**Published:** 2020-12-15

**Authors:** Cheng Zhang, Zuhua Chen, Xiaoyi Chong, Yang Chen, Zhenghang Wang, Ruoying Yu, Tingting Sun, Xiaoxi Chen, Yang Shao, Xiaotian Zhang, Jing Gao, Lin Shen

**Affiliations:** ^1^ Department of Gastrointestinal Oncology Key laboratory of Carcinogenesis and Translational Research (Ministry of Education/Beijing) Peking University Cancer Hospital & Institute Beijing China; ^2^ Department of Oncology Tongji Hospital Tongji Medical College Huazhong University of Science and Technology Wuhan China; ^3^ Translational Medicine Research Institute Geneseeq Technology Inc Toronto Ontario Canada; ^4^ Nanjing Geneseeq Technology Inc Nanjing China; ^5^ National Cancer Center/National Clinical Research Center for Cancer/Cancer Hospital & Shenzhen Hospital Chinese Academy of Medical Sciences and Peking Union Medical College Shenzhen China

**Keywords:** *ERBB2* copy number, gastric cancer, HER2‐targeted therapy, immunotherapy, liquid biopsy, TMB

## Abstract

**Background:**

Gastric cancer (GC) is confronted with limited options for precision medicine. Human epidermal growth factor receptor 2 (HER2) is the principal druggable target of GC, yet proper biomarkers for response/resistance prediction remain unveiled.

**Methods:**

From 40 GC patients received HER2‐targeted therapy, a total of 327 peripheral blood plasma specimens was collected including baseline and treatment time points. Circulating tumor DNA (ctDNA) was extracted and sequenced with a target panel of 425 genes. Experimental validation of resistant mutations was carried out in NIH‐3T3 cell line.

**Results:**

Genomic features, including *ERBB2* copy number variation (CNV), total copy number load, and tumor mutation burdens (TMBs), dynamically changed along with the treatment process and correlated with disease progression. Plasma ctDNA‐based diagnosis was more sensitive than conventional computed tomography scanning in 40% of investigated patients, gaining additional time for clinical management. Compared to baseline, new gene alterations were emerged in 12 patients who developed drug resistance during treatment. *ERBB2* mutations potentially related to Pyrotinib resistance were identified in plasma ctDNA of one patient and functional analysis of their downstream signaling pathways was carried out in NIH‐3T3 cell line. TMB exhibited more power than *ERBB2* CNV in predicting treatment responses and prognosis for HER2‐targeted therapy in GC patients. Interestingly, survival analysis indicated that patients harboring both HER2 (*ERBB2*) positivity and high TMB might gain more therapeutic benefits from immune checkpoint inhibitors instead of HER2‐targeted regimens that required further studies and validations

**Conclusions:**

Our work showed that the dynamic surveillance of plasma ctDNA genomic features provided instructive information for the precision medication of GC patients.

## Graphical abstract

- Plasma ctDNA‐based diagnosis was more sensitive than conventional computed tomography scanning in 40% of patients, gaining time for clinical management.
- Plasma tumor mutation burden was a potential marker to predict the therapeutic outcomes of human epidermal growth factor receptor 2 (HER2)‐targeted therapy in GC.
- HER2‐positive patients with high TMB might gain more therapeutic benefits from immunotherapy instead of HER2‐targeted regimens that required further studies and validations.

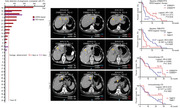

## 1 INTRODUCTION

Gastric cancer (GC) is one of the leading causes of cancer death around the world.^1^ As a digestive tract‐originated malignancy, GC is featured by its complicated tissue composition and high heterogeneity, which greatly hinder its early diagnosis and treatment.^2^ The recent development of precision medicine has put forth the hope to expanded survival expectance or even cure for multiple types of cancer, yet the individualized treatment for GC remains to be improved.

Human epidermal growth factor receptor 2 (HER2; encoded by *ERBB2*) overexpression and amplification have been recognized as frequent molecular abnormalities in GC.^3^, ^4^ Due to its tight regulation to crucial downstream signaling nodes such as RAS/RAF/MYC and PI3K‐Akt, HER2 remains to be the prior choice of targeted drug development for patients with advanced GC.^5^ To date, a variety of HER2‐targeted drugs has been developed and evaluated by clinical trials separately and combined, including HER2 antibody and its derivatives (Trastuzumab and Pertuzumab), tyrosine kinase inhibitor (Lapatinib, Neratinib, and Afatinib), and antibody‐drug conjugates (ADCs) (DS‐8201a, TDM1, and RC48).^6^, ^7^, ^8^, ^9^, ^10^, ^11^, ^12^, ^13^ Monoclonal antibody Trastuzumab is the only HER2‐targeted agent currently approved for first‐line practice of GC.^14^ DS‐8201a, also known as Trastuzumab deruxtecan, is an ADC conjugated with humanized anti‐HER2 antibody, cleavable peptide‐based linker, and topoisomerase I inhibitor payload. DS‐8201 has been evaluated in a phase II trial consisted of HER2‐positive advanced GC. Both response rate and overall survival (OS) was significantly improved in DS‐8201‐treated patients compared to standard therapy.^8^ TDM1(Trastuzumab emtansine) has shown to be active in HER2‐positive breast cancer patients with brain metastasis from the phase III KAMILLA trial.^13^ Another HER2‐ADC, RC48‐ADC, has been carried out in phase II trials and demonstrated a clinically meaningful overall response rate (ORR) in pretreated HER2‐positivie urothelial carcinoma patients.^15^ Although these agents/combinations provide supporting evidence for further investigation in HER2‐positivie GC, the response rate of HER2‐positive patients received HER2‐targeted therapy remain limited, and the frequently upcoming resistance is another reason that impaired therapeutic efficacy.

In order to overcome the unsatisfactory situation of HER2‐targeted therapy, timely evaluation of gene alterations along with the treatment progresses is of great importance so as to aid in better therapeutic management and clinical decision‐making for GC patients. However, conventional diagnostic methods, such as computed tomography (CT)‐scanned imaging and invasive endoscopic sampling, are insufficient to fulfill the need of real‐time disease surveillance. Furthermore, the genomic profiles and molecular traits of metastatic tumor tissue are sometimes inconsistent with the primary tumors, making it difficult to monitor the cancer progression on tissue level. As a type of tumor‐derived, fragmented cell free DNA, ctDNA (circulating tumor DNA) is enriched in blood (specifically, plasma) of cancer patients and reflecting the information of the entire tumor genome. By elucidating the genomic features in patient's plasma with next generation sequencing (NGS), ctDNA‐based liquid biopsy complements the shortages of tissue evaluation and has been proved to be a powerful choice to facilitate the monitoring of cancer progression.^16^, ^17^ ctDNA levels correlate with the tumor size, stage, and depth of invasion in patients with GC.^18^ Previous study showed that GC patients with higher ctDNA levels had higher risk of peritoneal recurrence and exhibited a 5‐year global survival rate poorer than patients without detected ctDNA.^19^ Meanwhile, the possibility of ctDNA monitoring in clinical response to immunotherapy and tracking HER2 resistance have been also been demonstrated in GC.^20^, ^21^ In this study, by evaluating the longitudinal changes of ctDNA in plasma derived from advanced GC patients during HER2‐targeted therapy, we seek to identify the genetic information that directed responses and prognosis, and to explore additional therapeutic options for advanced GC.

## 2 MATERIALS AND METHODS

### 2.1 Study design and specimen information

All patients in this study were enrolled and followed‐up by the Department of Gastrointestinal Oncology, Peking University Cancer Hospital & Institute for conventional therapy or clinical trials. Peripheral blood specimens were collected with cell‐free DNA BCT tubes (Streck Laboratories, USA). All clinical data of patients were acquired by referring to their individual medical records. For both cohorts, the inclusion criteria were as follows: patients must be diagnosed with unresectable or metastatic GC, had at least one measurable, or unmeasurable but evaluable, lesion (described according to RECIST 1.1), without history of severe heart or liver disease, psychiatric disorders, without hemorrhage or perforation of digestive tract, remained treatment naive 3 weeks before the baseline of treatment, scored with ECOG (Eastern Cooperative Oncology Group) performance status of 0/1 three days before the baseline of treatment. Exclusion criteria were as follows: patients harbored GC combined with other types of cancer, or histopathologically diagnosed as neuroendocrine tumor or squamous type of tumor.

Based on these criteria, 45 patients treated with anti‐HER2 regimens (including conventional treatment and clinical trials) during October 1, 2013 to October 1, 2018 were enrolled and 40 were included in the HER2‐targeted therapy cohort (Figure S1A). Totally, 327 peripheral blood samples from pretreatment baseline (BL) to disease progression were collected every two treatment cycles for the 40 GC patients. By referring to CT, patients’ responses to HER2‐targeted therapy were evaluated as partial response (PR), stable disease (SD), or progressed disease (PD) according to the RECIST 1.1 criteria, whereas no complete response (CR) was observed in this cohort. For each series of specimen, the time of the first response evaluation after treatment was defined as first point (FP). Additionally, 39 patients treated with immune checkpoint inhibitors (from clinical trials) during Jun 1, 2016 to Jun 1, 2020 were enrolled and 37 were included in the immunotherapy cohort (Figure S1B). Baseline blood specimens of these 37 GC patients received immunotherapy were collected for validation.

Written informed consents were obtained from all donors. These specimens and associated clinical information were approved for experimental applications by the Institutional Ethics Committee, Peking University Cancer Hospital & Institute. This study was conducted in accordance with the Declaration of Helsinki.

### 2.2 Plasma isolation and cfDNA extraction

Genomic DNA from white blood cells (WBCs) was extracted using DNeasy Blood & Tissue Kit (Qiagen, Germany) and used as normal control. After centrifuging whole blood samples at 4℃ for 10 min (1600 g), plasma supernatant was separated with blood cell sediment and then re‐centrifuged at 4℃ for 10 min (16 000 g). Plasma cfDNA was extracted using Qiagen QIAamp Circulating Nucleic Acid Kit (Qiagen, Germany) following the manufacturer's protocols, qualified with Nanodrop 2000 (Thermo Fisher Scientific), and quantified by Qubit 2.0 using the dsDNA HS Assay Kit (Life Technologies) according to the manufacturer's recommendations. The median yield for the plasma cfDNA was 25.9 ng, ranging from 10.1 to 739.2 ng. Isolated blood components (plasma, serum, and PBMC) and extracted cfDNAs were maintained at –80℃ for long‐term storage.

### 2.3 Next generation sequencing

NGS and data processing were performed as previously described.^21^ In brief, isolated ctDNA samples were processed with the KAPA Hyper Prep kit (KAPA Biosystems) for libraries construction. A customized NGS panel targeting 425 cancer‐relevant genes was used for hybridization enrichment. The list of panel genes was displayed in Table S2. Indexed DNA libraries were pooled together to a total amount of 2 μg and subjected to probe‐based hybridization using IDT xGen Lockdown reagents (IDT, Coralville, IA) and Dynabeads M‐270 (Thermo Fisher). The library was quantified by KAPA Library Quantification kit (KAPA Biosystems) according to manufacturer's instructions. Bioanalyzer 2100 (Agilent, USA) was used to determine the fragment size distribution of the final library and then sequenced on Illumina HiSeq4000 NGS (Illumina) platform following the manufacturer's instructions. An expected sequencing depth of 3000× was set for ctDNA samples.

### 2.4 Data processing

Trimmomatic was used for FASTQ file quality control. Leading/trailing low quality (quality reading below 30) or N bases were removed. Remaining reads were mapped to the reference sequence data (Human Genome version 19) using Burrows‐Wheeler Aligner (BWA‐mem, v0.7.12). Indels realignment and base quality score recalibration were performed with Genome Analysis Toolkit (GATK 3.4.0). Somatic mutations were detected with VarScan2. Copy number variations (CNVs) were detected using ADTEx (http://adtex.sourceforge.net) with default parameters as reported by previous studies.^21^, ^22^, ^23^ To eliminate sequencing artifact, a local bioinformatics pipeline was performed. First, we used a local WBCs database, containing recurrent somatic alterations from WBCs of 400 patients, to eliminate the sequencing artifacts. Specifically, if a variant was detected (i.e., more than mutant reads) in >10% of the samples, it was considered a likely artifact and was removed. Second, a background denoising strategy was performed. Briefly, we performed panel sequencing with similar sequencing depth used in this study on plasma samples of 50 healthy individuals to assemble a database of alterations at each site of the panel and build a background error model. A specific alteration at a specific site was considered sequencing noise if the allele frequency (AF) and distinct supporting reads were not significantly beyond the background error probability. Only alteration with an AF over three standard deviations from the mean AF of healthy plasma ctDNAs pool remained and subjected for further analyze.

The cutoffs of gene CNVs were set to 1.4‐fold copy number for amplification (gain) and 0.65‐fold copy number for deletion (loss). Total copy number load (TCL) was summarized by counting amplifications and deletions. Tumor mutation burden (TMB) was calculated by summing all base substitutions and indels in the coding region of targeted genes, including synonymous alterations to reduce sampling noise and excluding known driver mutations as previously described.^24^ Samples within the highest mutation load or CNV load tertile (top 33.3%) were classified as having high TMB or TCL, which equal to ≥8.5 mutations/Mb in TMB or ≥2 in TCL. Samples with <8.5 mutation/Mb in TMB or <2 in TCL were considered as low TMB or TCL. These cutoffs were consistently used for both the HER2‐targeted therapy and immunotherapy cohort. Neutrophil‐to‐lymphocyte (NLR) or monocyte‐to‐lymphocyte ratio (MLR) were defined as the absolute neutrophil/monocyte count divided by the absolute lymphocyte count.^25^, ^26^ Maximum variant allele frequency was defined as the highest allele frequency of the detected somatic variants in each sample.

### 2.5 Cell culture, transfection, and drug treatment

Mouse embryonic fibroblast cell line NIH‐3T3 was purchased from Cell Bank (Chinese Academy of Sciences, China). Cells were maintained in RPMI‐1640 medium (Invitrogen, Carlsbad, CA) supplemented with 1% penicillin plus streptomycin (HyClone, Logan, UT) and 10% fetal calf serum (Gibco BRL), and then incubated at 37℃ in a humidified incubator (5% CO_2_). Wild‐type and point mutation of *ERBB2* were constructed into plasmids and transfected into cells with Lipofectamine 3000 reagent (Thermo Fisher, USA). After 36 h of transfection, cells were treated with Pyrotinib (200 nm) for additional 48 h. Pyrotinib (SHR‐1258) was purchased from Selleck.

### 2.6 Western blot assay

For western blotting, cells were collected and lysed in 1× SDS‐PAGE loading buffer (1% SDS, 11% glycerol, 10% β‐mercaptoethanol, 0.1 M Tris, pH 6.8) for assay. Protein samples were then probed with corresponding primary/secondary antibodies, illuminated with the Clarity Western ECL substrate (Bio‐Rad, Hercules, CA), and visualized for protein bands with Amersham Imager 600 (GE Healthcare, Chicago, IL). Antibody for HER2 (#4290), p‐S6 (S240/S244, #4858), S6 (#2217), p‐Akt (S473, #9271), Akt (#4691), p‐Erk (T202/Y204, #4370), Erk (#4695), and β‐actin (#4970) were purchased from Cell Signaling technology.

### 2.7 Statistical analysis

Numerical diversity between subgroups was assessed by Wilcoxon‐Mann‐Whitney test. The consistency between ctDNA genomic variations and carcinoembryonic antigen (CEA) level was assessed by Spearman rank‐order correlation coefficient. Concordance between ctDNA *ERBB2* copy number and tissue HER2 expression was assessed by Kappa test. Pathway involvement and gene set enrichment were performed by referring to GO (http://geneontology.org/), DAVID (https://david.ncifcrf.gov/), and KOBAS (http://kobas.cbi.pku.edu.cn/) bioinformatics resources. Survival proportions were assessed by Kaplan‐Meier survival analysis paired with Log‐rank test. For all these tests, *P *< .05 was considered as statistically significant. All analyses were performed with SPSS 21.0 or R program, and then formatted with GraphPad Prism 6.

## 3 RESULTS

### 3.1 Overall characteristics of enrolled patients

Forty GC patients received HER2‐targeted therapy or HER2‐targeted therapy plus chemotherapy were enrolled in this study, with a median age of 59 (29‐75 years). Thirty‐one patients (77.5%) were male and nine (22.5%) were female. Most patients (85%; 34/40) were non‐esophagogastric junction origination, diagnosed with middle (50%; 20/40) or low (45%; 18/40) differentiation, or classified as intestinal subtype (70%; 28/40). Confirmed by immunohistochemistry (IHC) staining or fluorescence in situ hybridization (FISH), 77.5% (31/40) patients were HER2 positive, whereas the rest were recruited by HER2‐targeted clinical trials. According to regimens, 14 patients received Trastuzumab‐combined chemotherapy (Paclitaxel, Oxaliplatin, Fluorouracil, etc) treatment, 11 received the JACOB treatment (Pertuzumab plus Trastuzumab‐combined chemotherapy), eight received HER2‐targeted antibody‐drug conjugate (ADC) treatment (TDM1 or RC48), and seven received HER2‐targeted inhibitor treatment (Pyrotinib or Pirotinib). Additionally, seven patients received two lines of HER2‐targeted treatments.

In total, ORR, disease control rate (DCR), and median progression‐free survival (mPFS) for the cohort were 50%, 75%, and 4.4 months, respectively. The PFS of the first, second to third line HER2‐targeted treatment was shown in Figure S2A. Patients in JACOB treatment displayed better efficacy than Trastuzumab‐combined chemotherapy, ADC, or inhibitors (Figure S2B), which is complied with current clinical consensus. The clinicopathological features of all patients and specimens were concluded as Table S1.

### 3.2 Genomic features in ctDNA dynamically reflected treatment response during HER2‐targeted therapy

**FIGURE 1:**
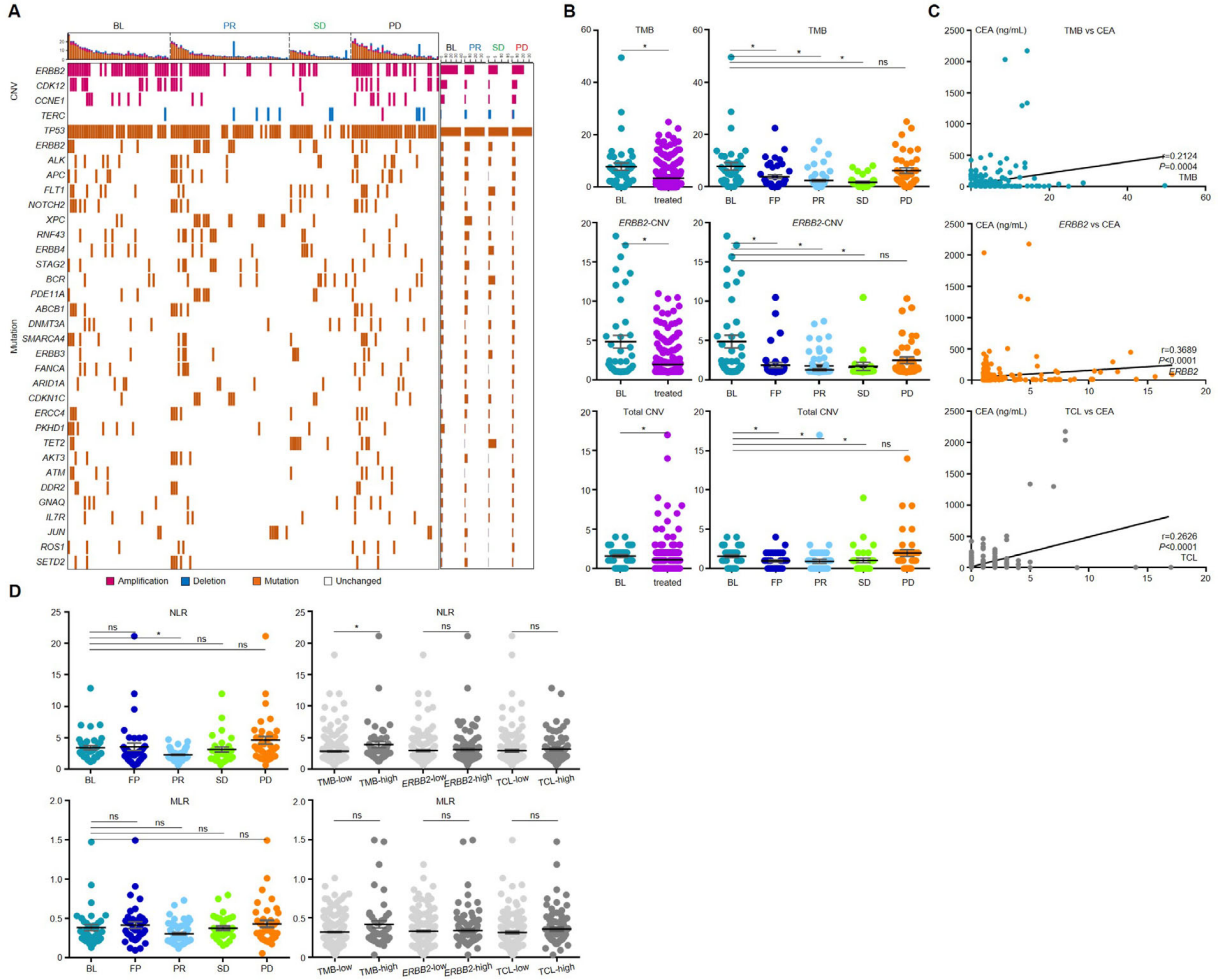
ctDNA genomic features dynamically changed in gastric cancer patients received HER2‐targeted therapy. **(A)** The landscape for all specimens collected at BL, PR, SD, and PD. **(B)** Comparison of *ERBB2* copy number, total copy number load (TCL), and tumor mutation burden (TMB) across all evaluation points. **(C)** The Spearman correlation of *ERBB2* copy number, TCL, and TMB with CEA. **(D)** The NLR/MLR values across all evaluation points and groups with low‐ or high‐ctDNA genomic features. *P < .05. ns, not significant

For the 40 enrolled patients, ctDNAs were extracted from 327 plasma samples and sequenced by NGS. Genomic features of 425 genes, including CNVs and mutations, were evaluated along with the proceeding of treatment. Genomic landscape with evaluated responses (BL, PR, SD, and PD) is shown in Figure 1A. The most frequently observed copy number changes were in *ERBB2/CDK12*/*CCNE1*/*TERC*, whereas the top 10 somatic mutations were observed in *TP53*/*ERBB2*/*ALK*/*APC/FLT1/NOTCH2/XPC*/*ERBB4*/*STAG2/BCR* (Figure 1A). The trends of *ERBB2* copy number, TCL, and TMB changes in each individual case during HER2‐targeted treatment were displayed in Figure S3.

We stratified all plasma samples according to time point and compared *ERBB2* CNV, TCL, and TMB status. As shown in Figure 1B, the level of ERBB2 CNV, TCL, and TMB all reduced after treatment. The reduction began at the first point after treatment (FP), reached the bottom at PR, remained low at SD, and recovered to the similar levels as BL at PD, suggesting that tumor fraction in plasma ctDNA was efficiently eliminated by HER2‐targeted treatment, yet recovered along with the progression of disease.

The correlation between these genomic features and other blood indexes were also investigated. Levels of *ERBB2* copy number, TCL, and TMB were positively correlated with the tumor biomarker CEA (Figure 1C). For immune‐cell‐related indexes, the NLR was reduced at PR, yet was recovered or even elevated at PD (Figure 1D). Combining samples from different time point, NLR was only significantly higher in TMB^high^ compared to TMB^low^ (Figure 1D). On the other hand, MLR displayed similar trend of dynamic changes to NLR, yet was insignificantly differed across all time points or genomic feature‐based classifications.

### 3.3 ctDNA shared a high concordance with tissue assay in identifying *ERBB2*/HER2 positivity

The *ERBB2*/HER2 status evaluated by ctDNA (NGS) and tissue assay (including IHC and FISH) displayed a high consistency. Eighteen patients had biopsied tumor tissue and matched plasma at BL (Table S3), among which a significant concordance of 94.4% (17/18) was achieved (Table S4), verifying that plasma *ERBB2* could be used as an alternative method for the screening of HER2‐targeted population. Notably, for the patient (case 39) displayed inconsistency between pathological diagnosis and ctDNA assay, the record of IHC staining was negative while *ERBB2* copy number was 1.7937. This inconsistency could be due to that plasma *ERBB2* copy number was slightly higher than the cutoff of amplification (1.4), or that the section of tissue containing amplified *ERBB2* was not captured by biopsy.

### 3.4 Genomic features in ctDNA forecasted disease progression earlier than CT scanning

**FIGURE 2:**
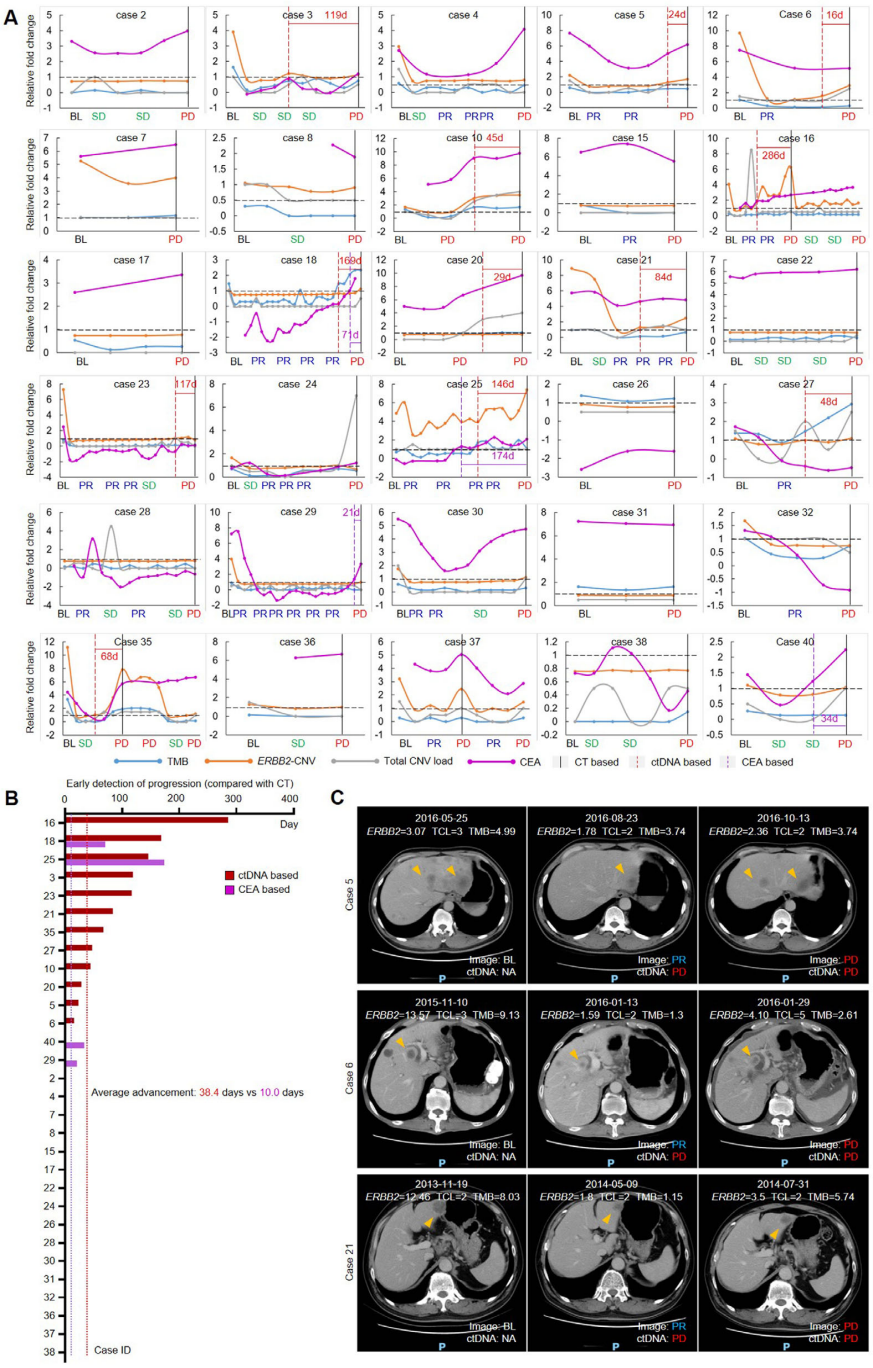
Genomic features in ctDNA facilitated the early detection of disease progression during HER2‐targeted therapy. (**A)** The real‐time changes of ctDNA features (*ERBB2* copy number, total copy number load [TCL], and tumor mutation burden [TMB]) and CEA for 30 patients who ever reached progressed disease (PD). Computed tomography (CT)‐based PD, ctDNA‐based PD, and CEA‐based PD were marked by separate vertical lines. **(B)** The advancement of ctDNA‐based PD and CEA‐based PD compared with CT‐based PD in detecting disease progressions for the 30 patients received HER2‐targeted therapy. **(C)** CT‐scanned images for three representative cases were displayed to compare ctDNA‐based PD with CT‐based PD

Current evaluation standard of therapeutic response (the RECIST guidance) relies on CT scanning, which was economically inefficient for dynamic detection and usually missed occult lesions, especially in the abdominal region. To compare the plasma ctDNA testing and CT scanning in monitoring disease progression, patients who reached disease progression by CT and possessed at least three plasma specimens were used for analysis. Dynamics of the three genomic features (*ERBB2* copy number, TCL, or TMB levels) along with the treatment were normalized to relative fold changes by comparing to their respective cutoff values. If either of genomic features elevated beyond the respective cutoff for at least two time points, the first point was considered as ctDNA‐based PD (Figure 2A). According to this standard, ctDNA‐based PD was detected ahead of CT‐based PD in 12 of 30 (40%) cases with an average advancement of 38.4 days (Figure 2B). Representative images for ctDNA‐based and CT‐based PD were demonstrated (Figure 2C).

Additionally, we also assessed whether changes of CEA exerted similar function to forecast disease progression. Concentrations of CEA were normalized to standard cutoff value (5 ng/mL) and were displayed in diagram as log2‐transformed indexes (Figure 2A). CEA‐based PD was detected ahead of CT‐based PD in four of 30 cases (13.3%) case with an average advancement of 10.0 days (Figure 2B), evidently less efficient than ctDNA‐based PD. Our data indicated that plasma ctDNA‐based liquid biopsy may serve as a complementary test to aid in the disease monitoring for GC patients, so as to gain further time for clinical practices.

### 3.5 Emerging genomic aberrations identified in ctDNA potentially contributed to therapeutic resistance

**FIGURE 3:**
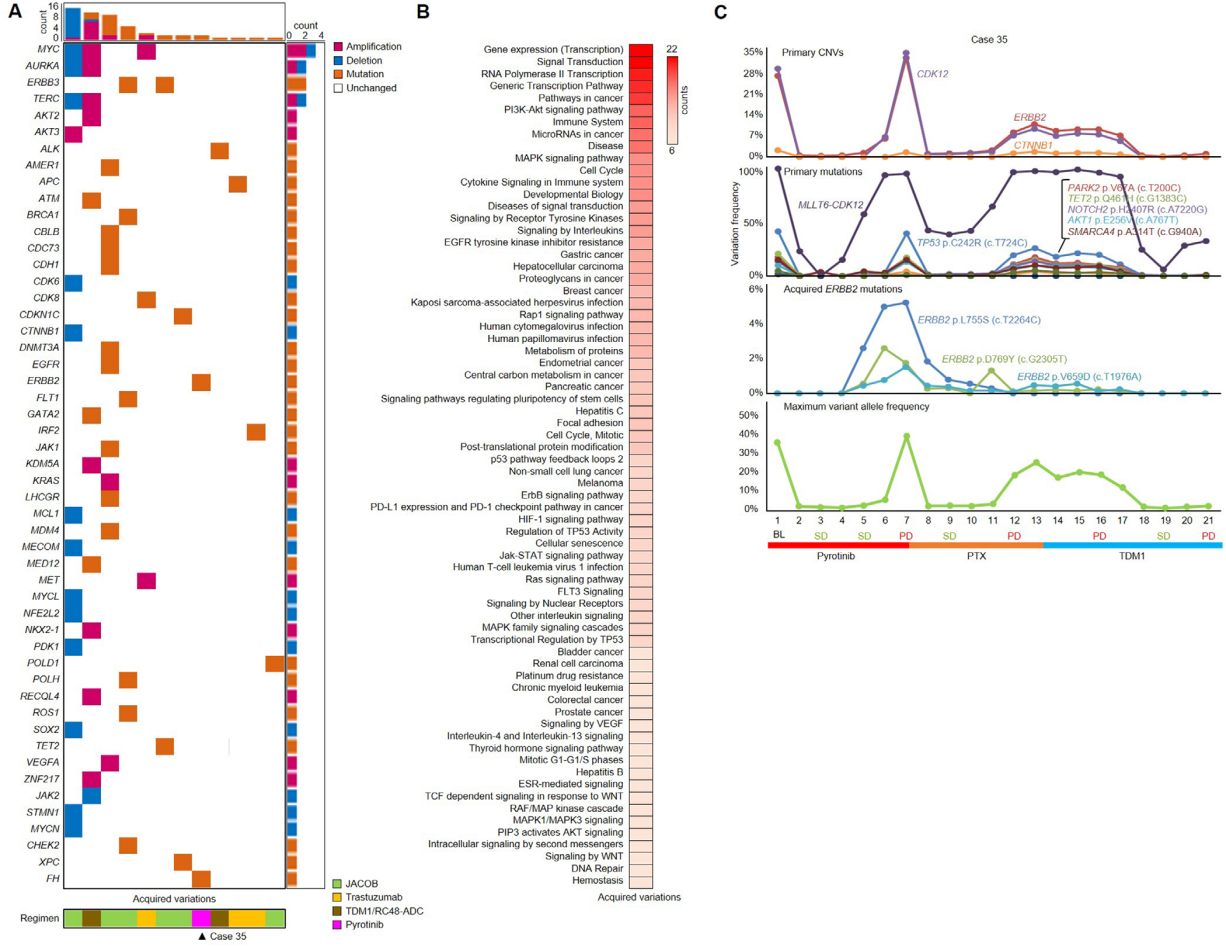
Emerging genomic aberrations potentially contributed to gastric cancer's (GC) resistance to HER2‐targeted therapy. **(A)** The distribution of novel gene alterations emerged in 12 patients and their correlation with HER2‐targeted regimens. **(B)** The biological processes and pathways involved with the emerging genomic aberrations were enriched. **(C)** The real‐time frequency of primary copy number variations (CNVs), primary mutations, three novel *ERBB2* point mutations, and maximum variant allele frequency changed along with the treatments in case 35. PTX, paclitaxel

In our cohort, new genomic aberrations (compared with BL) were detected in 12 patients after developing therapeutic resistance (Figure 3A). The change of maximum variant allele frequency (MaxiVAF) in each sample during anti‐HER2 therapy from 12 patients with newly identified mutations was shown in Figures S2C and S2D. Here, we employed MaxiVAF as an indicator for the tumor fraction in the plasma ctDNA based on previous publication.^27^ As shown in Figure S2, there was no significant difference in MaxiVAF between baseline and PD samples, suggesting the newly identified mutations were likely acquired during anti‐HER2 therapy. These aberrations were vigorously annotated to multiple *ERBB2* downstream pathways (such as PI3K‐Akt and MAPK signaling) for patients received JACOB or Trastuzumab‐combined chemotherapy, or annotated to scattered cancerous pathways for patients received TDM1 or RC48‐ADC (Figures 3A and 3B). Patients without newly identified aberrations during treatment displayed slightly higher therapeutic responses (ORR = 52.2% vs 41.7%, DCR = 74% vs 66.7%) and slightly longer PFS (Figure S4) than patients with.

Notably, three *ERBB2* mutations (p.V659D [c.T1976A], p.L755S [c.T2264C], and p.D769Y [c.G2305T]) were identified in the plasma of case 35 after developing resistance to Pyrotinib treatment (Figure 3C). To find out whether these mutations were associated with Pyrotinib resistance, we transfected plasmids expressing these *ERBB2* mutations into NIH‐3T3 cell to study their impact on downstream molecules. Before Pyrotinib treatment, the phosphorylation of Erk (p‐Erk) was higher in three *ERBB2*‐mutation groups than in *ERBB2* wild‐type group, whereas phosphorylation of Akt and S6 was comparable across these groups. After administration of Pyrotinib (200 nm, 48 h), p‐Akt was unaffected, S6 was evidently repressed in both *ERBB2* mutation and wild‐type groups, and p‐Erk remained high for *ERBB2* p.V659D and p.L755S mutation groups (Figure S5), suggesting that Erk phosphorylation and activation induced by specific *ERBB2* mutations might be associated with resistance to Pyrotinib.

### 3.6 Plasma‐borne genomic features prior to treatment predicted responses to HER2‐targeted therapy

**FIGURE 4:**
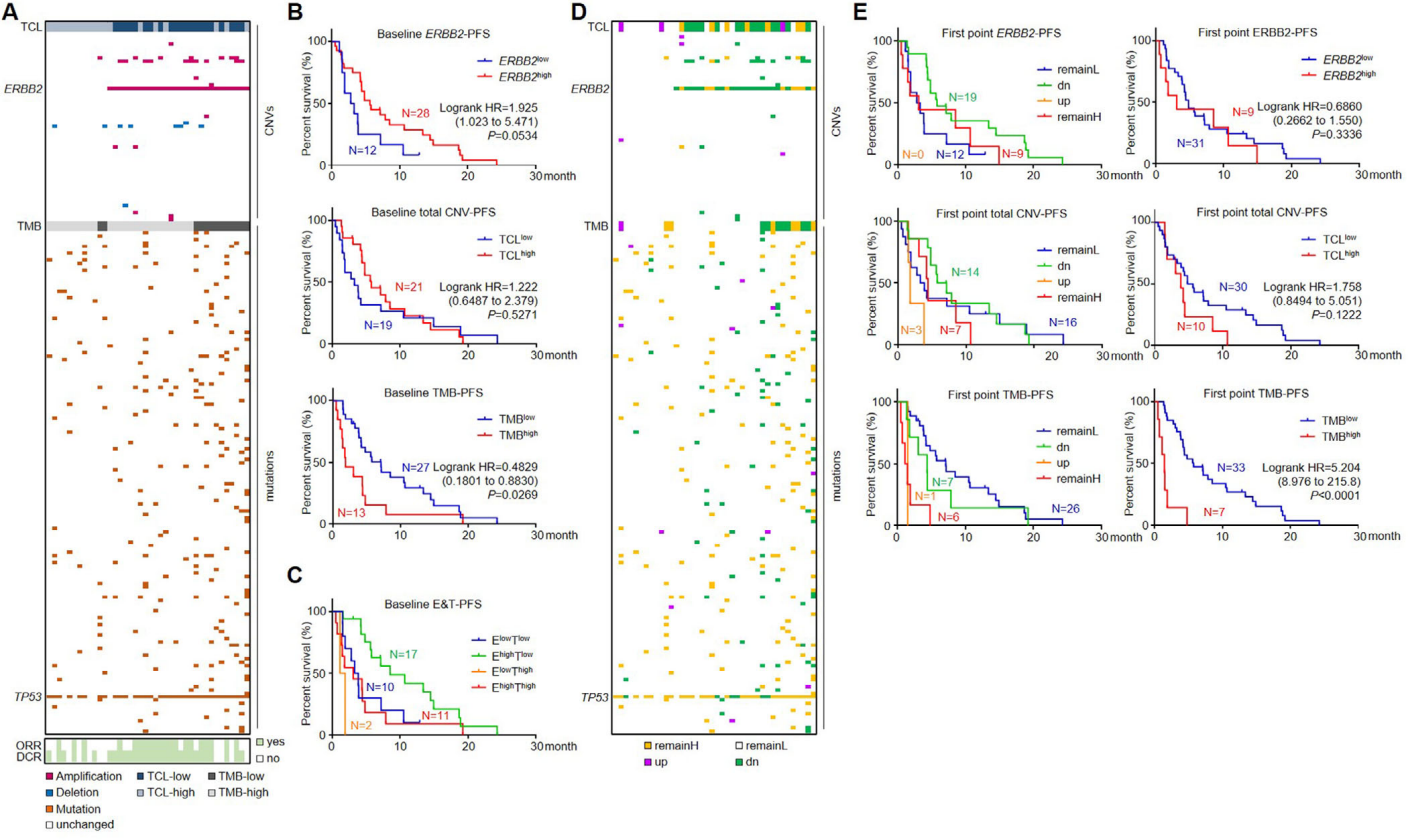
Genomic features served as predictive biomarkers for output of HER2‐targeted therapy. **(A)** An overview of *ERBB2* copy number, total copy number load (TCL), and tumor mutation burden (TMB) for 40 patients at baseline and individually paired responses. **(B)** *ERBB2* copy number, TCL, and TMB information at baseline were used to predict progression‐free survival (PFS) proportions of subsequent HER2‐targeted therapy. **(C)** PFS was calculated for patients stratified by baseline *ERBB2* copy number and TMB. **(D)** The relative changes of *ERBB2* copy number, TCL, and TMB for 40 patients at the first point after treatment started were compared with baseline status. **(E)** Stratified (left panel) or combined (right panel) information of *ERBB2* copy number, TCL, and TMB at the first point were used to predict PFS proportions of HER2‐targeted therapy. E^low^T^low^, *ERBB2* ^low^TMB^low^; E^high^T^low^, *ERBB2* ^high^TMB^low^; E^low^T^high^, *ERBB2* ^low^TMB^high^; E^high^T^high^, *ERBB2* ^high^TMB^high^

We then evaluated the potential of using ctDNA genomic information in guiding HER2‐targeted GC therapy. The genomic landscape at BL is shown in Figure 4A. We analyzed the genomic diversity between patients harboring different *ERBB2* or TMB status and performed pathway enrichment for each fraction (Figures S6A and S6B). Genomic aberrations in *ERBB2*‐high or TMB‐high patients involved in much more cancerous processes and pathways than *ERBB2*‐low or TMB‐low patients, whereas the *ERBB2*‐related comparison displayed larger disparity than TMB‐related comparison, emphasizing the validity of utilizing HER2‐targeted regimens in our cohort.

At baseline point, 28 cases carried *ERBB2* amplification (defined as *ERBB2* ^high^), with ORR = 60.7% (17/28) and DCR = 85.7% (24/28); 12 cases carried no *ERBB2* amplification (*ERBB2* ^low^), with ORR = 25% (3/12) and DCR = 50% (6/12), suggesting that although responded less vigorously than HER2 positive patients, a part of HER2 negative patients was also sensitive to HER2‐targeted therapy. Considering TCL, 21 cases were defined as TCL^high^, with ORR = 61.9% (13/21) and DCR = 90.5% (19/21); 19 cases were defined as TCL^low^, with ORR = 36.8% (7/19) and DCR = 57.9% (11/19). The high similarity between *ERBB2* copy number‐ and TCL‐predicted ORRs/DCRs indicated that *ERBB2* copy number might be the major contributor to therapeutic responses for all CNVs. From the view of TMB, 13 cases were defined as TMB^high^, with ORR = 38.5% (5/13) and DCR = 53.8% (7/13); 27 were defined as TMB^low^, with ORR = 55.6% (15/27) and DCR = 85.2% (23/27), suggesting that TMB^low^ patients were more likely to benefit from HER2‐targeted therapy (Figure 4A). We then further classified the 40 patients into four categories by combining baseline *ERBB2* and TMB status. For *ERBB2* ^high^TMB^high^ patients (11 cases), ORR and DCR were 45.5% (5/11) and 63.6% (7/11) , respectively; for *ERBB2* ^high^TMB^low^ patients (17 cases), ORR and DCR were 70.6% (12/17) and 100% (17/17) , respectively; for *ERBB2* ^low^TMB^high^ patients (two cases), ORR and DCR were 0% (0/2) and 0% (0/2) , respectively; and for *ERBB2* ^low^TMB^low^ patients (10 cases), ORR and DCR were 30% (3/10) and 60% (6/10), respectively. All above response rates are concluded in Table S5.

Kaplan‐Meier survival analysis was then performed to describe the longevity of response stratified by genomic traits. At baseline, high ERBB2 CNV and TCL status were not associated with better survival rate. Interestingly, TMB^high^ at baseline was associated with poorer PFS than TMB^low^ (Figure 4B). This prognostic inconformity between *ERBB2* and TMB indicated that HER2‐positive patients harboring high TMB may not benefit from HER2‐targeted therapy as much as expected. When combining these two parameters for stratification, *ERBB2* ^high^TMB^low^ patients displayed the highest response rate and the most favorable PFS; *ERBB2* ^low^TMB^high^ patients had the worst; and PFS of *ERBB2* ^high^TMB^high^ and *ERBB2* ^low^TMB^low^ patients lie in between (Table S5 and Figure 4C), suggesting that the combination of plasma‐borne *ERBB2* copy number and TMB was a powerful option to predict patients’ response to HER2‐targeted anti‐GC therapy. It is worth mentioning that although the prognostic role of TMB was observed only in patients receiving second or higher lines of HER2‐targeted therapy, PFS of *ERBB2* ^high^TMB^low^ group was slightly better than *ERBB2* ^low^TMB^high^ group for patients receiving first line HER2‐targeted therapy. However, due to the limited number of patients in each group, further study is warranted to validate this observation (Figure S7).

### 3.7 Plasma‐borne TMB dynamically surveillant therapeutic outputs of HER2‐targeted therapy

Considering that ctDNA genomic traits dynamically changed along with HER2 targeted therapy, we investigated whether changes in plasma at the first point (FP) after treatment started provided useful information in therapeutic surveillance. Genomic features at FP were compared with those at BL. According to changes of *ERBB2* copy number/TCL/TMB, patients were stratified into four categories: dn (from AMP/high at BL to non‐AMP/low at FP), up (from non‐AMP/low at BL to AMP/high at FP), remainL (non‐AMP/low at both BL and FP), and remainH (AMP/high at both BL and FP). Relative changes of these genomic features are shown as Figure 4D.

For all 40 patients at FP, considering *ERBB2* copy number, *ERBB2*‐dn cases possessed higher ORR and DCR (63.2% and 94.7%, respectively) than *ERBB2*‐remainH (55.6% and 66.7%, respectively) and *ERBB2*‐remainL (25% and 50%, respectively) cases, whereas no patients were defined as *ERBB2*‐up; considering TCL, TCL‐dn cases possessed higher ORR and DCR (64.2% and 92.9%, respectively) than TCL‐remainH (57.1% and 85.7%, respectively), TCL‐remainL (43.8% and 62.5%, respectively), and TCL‐up (0% and 33.3%, respectively) case; considering TMB, TMB‐remainL cases possessed higher ORR and DCR (57.7% and 88.5%, respectively) than TMB‐dn (42.9% and 71.4%, respectively), TMB‐remainH (33.3% and 33.3%, respectively), and TMB‐up (0% and 0%, respectively) case. In general, patients with reduced or remained‐low TMB at FP were better responders to HER2‐targeted therapy, whereas changes of *ERBB2* and TCL at FP were less efficient in predicting therapeutic responses. All these response rates are concluded in Table S6.

We then performed Kaplan‐Meier analysis for those categories at FP. For *ERBB2* stratification, PFS of *ERBB2*‐dn patients were slightly longer than *ERBB2*‐remainL or *ERBB2*‐remainH patients, suggesting a favorable prognostic trend to HER2‐targeted regimens. For TCL stratification, TCL‐dn/TCL‐remainL patients had the longest and TCL‐up patients had the shortest PFS. For TMB stratification, TMB‐remainL and TMB‐dn patients had longer PFS than TMB‐remainH and TMB‐up patients (Figure 4E, left panel). We further combined all groups of “up” with “remainH” and “dn” with “remainL” into a two‐dimensional stratification manner. Status of *ERBB2* and TCL did not correlate with PFS, whereas high level of TMB predicted a poor PFS (Figure 4E, right panel). Combining both BL and FP conditions, we concluded that compared with *ERBB2* copy number or TCL, plasma TMB was a better option to predict and dynamically surveillant the therapeutic outputs of HER2‐targeted therapy in GC.

### 3.8 Immunotherapy may serve as a better therapeutic option than HER2‐targeted therapy for *ERBB2* ^high^TMB^high^ GC patients

**FIGURE 5:**
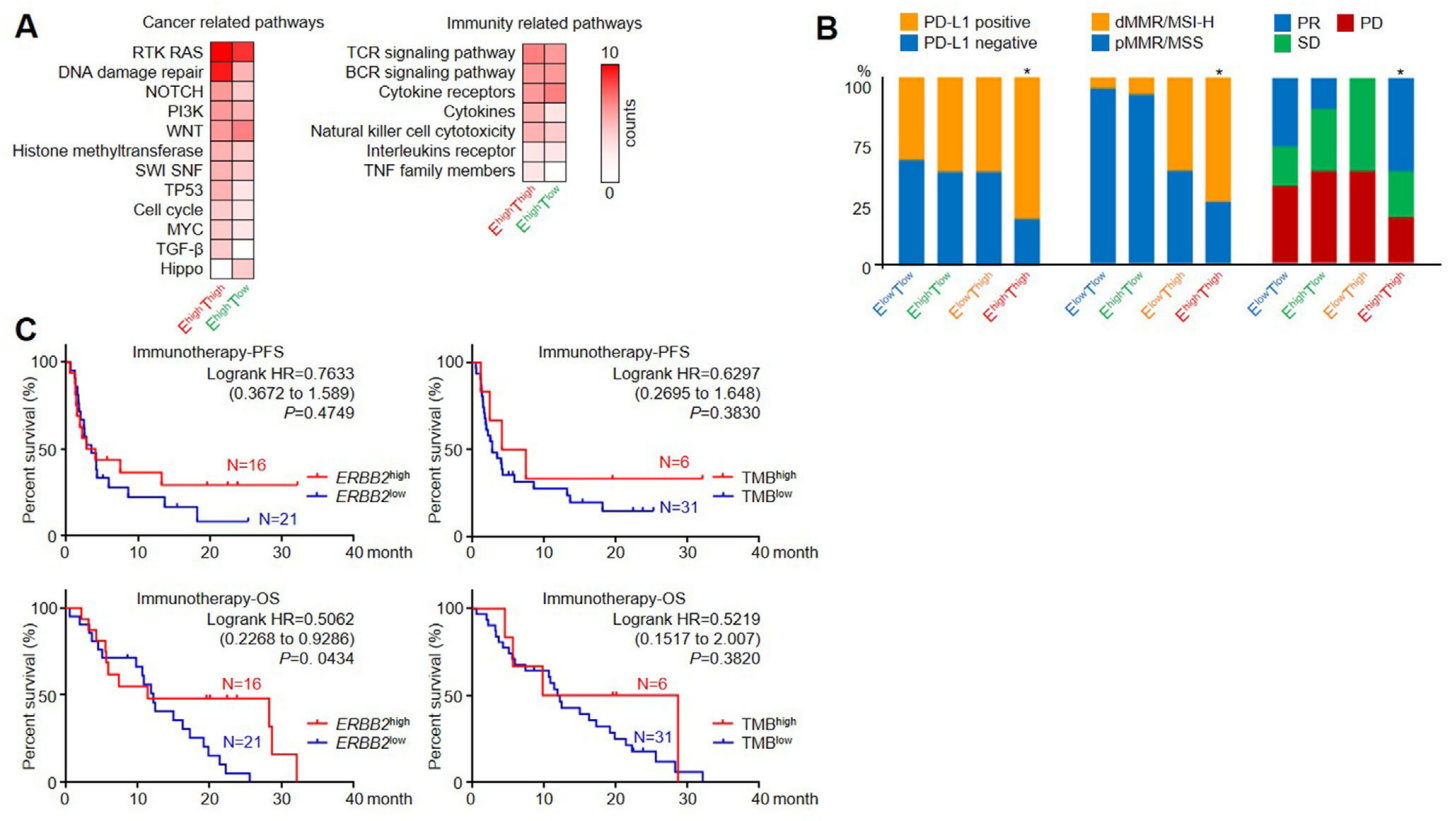
*ERBB2* ^high^TMB^high^ patients displayed a more favorable outlook for immunotherapy. **(A)** Through investigating the ctDNA genomic changes in the HER2‐targeted cohort, the major cancer‐related and immunity‐related pathways displayed a higher enrichment in the *ERBB2* ^high^TMB^high^ group. For the immunotherapy cohort, **(B)** the correlation of PD‐L1 expression, MSI status, and responses after received immune checkpoint inhibitors were compared across *ERBB2*‐TMB‐stratified patients. **(C)** Progression‐free survival (PFS) and overall survival (OS) for patients with different *ERBB2*/TMB status were demonstrated

As we have demonstrated, *ERBB2* ^high^TMB^high^ patients were unlikely to benefit much from HER2‐targeted therapy if they carried high TMB (Table S5 and Figure 4C). To mark a mechanistic insight, we performed enrichment analysis for the pathways potentially disturbed by plasma‐borne genomic aberrances. The numbers of enriched signaling were larger in *ERBB2* ^high^TMB^high^ than in *ERBB2* ^high^TMB^low^ patients at BL (272 vs 17) and FP (195 vs 58). Combining BL and FP, the most enriched pathways were displayed in Figure 5A. Apart from several major cancer‐related pathways, immunity‐related pathways were also found more enriched in *ERBB2* ^high^TMB^high^ patients. The enrichment diversity between these two subgroups suggested that accumulation of tumor mutations dysregulated cancerous signaling and disrupted tumor immune responses.

We then explored whether these HER2‐positive/*ERBB2* amplified plus TMB‐high patients were apt for immunotherapy. We referred to an immunotherapy cohort consisted of 37 GC patients recruited by PD‐1/PD‐L1/CTLA‐4‐targeted clinical trials (patient information is shown in Table S7). The ORR and DCR for the immunotherapy cohort were 29.7% (11/37) and 56.8% (21/37), respectively, inferior to the HER2‐targeted cohort (50% and 75%), whereas mPFS and mOS were 3.53 and 11.9 months, respectively. In this immunotherapy cohort, ctDNAs for baseline samples were extracted and sequenced for *ERBB2* copy number and TMB. With the same cutoff standard as in the HER2‐targeted cohort, 12 TMB^low^ *ERBB2* ^high^ and four TMB^high^ *ERBB2* ^high^ patients were identified.

**FIGURE 6:**
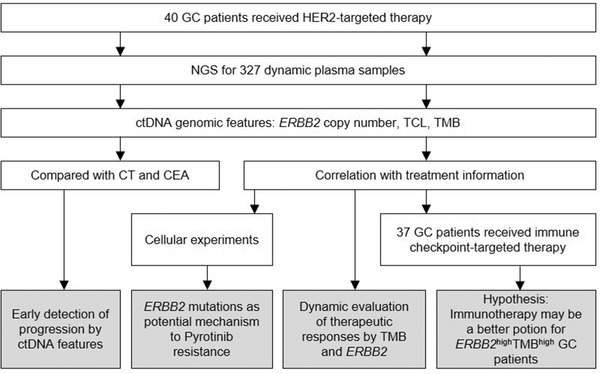
An overview of the workflow and the major conclusions in this study. Through tracking the serial changes of plasma ctDNA from GC patients underwent HER2‐targeted therapy or immunotherapy, our work provided four section of clues that directed the clinical decision‐making of GC

The positive rates for PD‐L1 (75% vs 50%) and dMMR/MSI‐H (66.7% vs 9.1%) were higher in *ERBB2* ^high^TMB^high^ patients than in *ERBB2* ^high^TMB^low^ patients (Figure 5B). However, PFS and OS showed no significant differences between TMB^high^ and TMB^low^ group, which might due to the limited numbers of patients. *ERBB2* ^high^ patients showed a better OS but not PFS than *ERBB2* ^low^ patients (Figure 5C). Additionally, *ERBB2* ^high^TMB^high^ patients displayed higher response rates (ORR = 50% vs 16.7%; DCR = 75% vs 50%) and a trend of longer PFS/OS than *ERBB2* ^high^TMB^low^ patients after receiving immune checkpoint inhibitors (Figure S8). Compared with the previous HER2‐targeted cohort, the mPFS of *ERBB2* ^high^TMB^high^ patients received immune checkpoint inhibitors (19.85 month) was much longer than received HER2‐targeted regimens (3.07 month), whereas the mPFS of *ERBB2* ^high^TMB^low^ patients received immune checkpoint inhibitors (2.53 month) was inferior to patients received HER2‐targeted regimens (8.5 month). The overall workflow and major findings in this study are summarized in Figure 6.

## 4 DISCUSSION

GC remains to be one of the most prevalent and deadly types of cancer around the world. Due to the high heterogeneity and cavitary characteristic of the digestive tract, the development of precision medicine in GC lag behind other cancer types. Here, we analyzed plasma‐borne genomic information throughout the whole treatment processes and discovered that *ERBB2* copy number and TMB showed strong correlation with responses to HER2‐targeted therapy. As shown by our data, patients with high ctDNA *ERBB2* copy number at baseline exerted better response to HER2‐targeted therapy, which complied with other reported studies based on tissue HER2 positivity.^14^, ^28^ However, the reduction of *ERBB2* copy number at the first point after regimen administration was insufficient to mark an optimistic therapeutic output. On the other hand, patients with high TMB at baseline or first point both displayed poor response, suggesting that ctDNA‐derived TMB was a more powerful prognostic biomarker than *ERBB2* copy number. It is worth to be mentioned that 62% of patients received combination of chemotherapy and HER2‐targeted therapy. Chemotherapy has been demonstrated to lead to ctDNA reduction in GC,^29^ thus may have some impact on our interpretation of ctDNA analysis in this study.

Apart from tumor mutations, the accumulation of CNVs could also lead to cancer development and therapeutic resistance. TCL was reported to be inversely correlated with prognosis in isocitrate dehydrogenase (IDH)‐mutant astrocytoma,^30^ whereas the total CNV burden in metastatic prostate cancer was significantly higher than that in nonmetastatic prostate cancer.^31^ CNV burden was also considered as a pan‐cancer prognostic factor associated with recurrence and death.^32^, ^33^ However, the prognostic efficiency of TCL was inferior to TMB and *ERBB2* copy number in our cohort. We inferred that the intrinsic genomic heterogeneity of GC as well as the limited information provided by panel‐sequencing compared to whole exome/genome sequencing might have some impact on the precise evaluation of overall CNVs in the plasma ctDNA. The detectability of mutations that is highly affected by the plasma‐ctDNA frequency abundance is another factor that may impact TMB interpretation. The mutations detected in plasma ctDNA sometimes underrepresent the mutations harbored in tumor tissue, which leads to inaccurate TMB calculation. Thus, plasma TMB need to be interpreted with caution in clinical setting when identifying patients for better therapeutic outcomes. Nevertheless, because several studies have already explored the possibility to accurately estimate TMB from target sequencing using various gene panel across different cancer type,^34^, ^35^, ^36^ the relatively small panel size (425 candidate genes) of targeted sequencing does not discolored the findings and conclusions concerning TMB in our study.

HER2 is the most important therapeutic target in GC. Around 10‐20% GC patients are identified as HER2 positive, for whom Trastuzumab plus chemotherapy is the only regimen approved as the first‐line treatment.^37^ In order to expand the armory of therapy against GC, the development of additional HER2‐targeted compounds and regimens so as to dealing with emerging Trastuzumab tolerance/acquired resistance is an urgent need. Besides patients accepting conventional Trastuzumab‐based therapy, those who were administrated with other HER2‐targeted regimens (JACOB, TDM1, RC48‐ADC, and Pyrotinib) initiated by our center were also included in this study.^28^ Considering the limited case number (40 in total) and complicated therapeutic background (several patients were treated twice with HER2‐targeted regimens), we did not perform further stratification based on these regimens, but still, a consistent trend of mutation and copy number changes has been observed across all cases. On the other hand, given that this cohort is mainly consisted of HER2‐positive cases, the conclusion and observation we found in this study may not be applied to all GC cases. Meanwhile, this cohort bias may also affect the concordance with tumor tissue analysis of GC in other study, due to which limited HER2‐negative cases were included.

IHC staining combined with FISH of tissue has been recognized as the golden standard in identifying GC HER2 positivity.^38^ However, the requirement for formalin‐Fixed Paraffin‐Embedded (FFPE)‐embedded tissue slides overwhelms tissue‐based assay for quick diagnosis and dynamic surveillance of disease progression. In the era of precision medicine, omics technique‐powered liquid biopsy is being embraced for guiding therapeutic decisions and monitoring relapse. For example, nonsmall cell lung cancer patients tested positive for plasma EGFR mutation have been approved eligible for EGFR tyrosine kinase inhibitors.^39^ On the other hand, a study (TARGET) that aimed to allocate patients with advanced cancer to suitable clinical trials based on ctDNA assay also showed high concordance with tissue on aspects of both testing and therapeutic outcomes.^40^ Nevertheless, for GC, although several studies have reported liquid biopsy's efficacy in detecting disease and tracking therapeutic responses,^41^, ^42^, ^43^ the longitudinal plasma‐borne genomic details along with HER2‐targeted therapy remain largely uninvestigated. Our data provided clues that ctDNA copy number‐defined *ERBB2* positivity shared a high concordance with HER2 expression‐ or *ERBB2* copy number‐defined positivity in paired tissues, which emphasized the potential to utilize ctDNA assay as an auxiliary diagnosis and an alternative method in noninvasive, dynamic evaluation. On the other hand, although it has not been evaluated in this study, NGS‐quantified plasma‐borne TMB (pTMB or bTMB) status has been proved to be correlated with tissue TMB status, suggesting that plasma‐based NGS sequencing is feasible for TMB estimation and subsequent clinical evaluation.^44^, ^45^

CT scanning serves as the most common noninvasive method to diagnose and evaluate cancer progression.^46^ Originated from cavitary organs, gastrointestinal cancers involve in abdominal region and are generally highly metastatic. Therefore, the detection of lesions by CT has a low sensitivity in gastrointestinal cancer than in other solid tumors and accompanied with time delays.^47^ Although NGS‐based liquid biopsy for ctDNA has been reported as a powerful tool in screening, detecting, and monitoring cancer development,^48^ the advantage of liquid biopsy in predicting therapeutic responses is not well characterized yet. Our study demonstrated that plasma ctDNA‐based PD was able to be detected more than a month earlier than the conventional CT scan in 40% of GC patients, saving enough time for clinical decisions. However, there were some PD patients who were not covered by plasma ctDNA testing in this cohort, suggesting ctDNA may serve as a complementary rather than substitute test.

It has been pointed out by several studies that mutations of ctDNA *ERBB2* correlated with HER2‐targeted resistance in cancer. *ERBB2* L869R mutation was reported to contributed to Trastuzumab resistance in breast cancer.^49^ *ERBB2* S310F, S310Y, and E321G mutants exhibited poor response to Trastuzumab in lung cancer.^50^ Previous reports indicated that D769Y was an activation mutation form of HER2,^51^ whereas L755S was known to confer Lapatinib resistance.^52^ Our findings indicated that specific *ERBB2* mutations (V659D and L755S) might contribute to acquired resistance to Pyrotinib therapy, yet further studies are warranted to confirm these results and to reveal the phenotypical changes induced by these mutations.

In addition to prompting early diagnosis and pre‐imaging evaluation, optimizing other regimens for patients is also a key point in improving therapeutic efficiency. By targeting immune‐suppressive checkpoint molecules (e.g., PD1, PD‐L1, and CTLA‐4), immunotherapy rescued patients’ immune system and prevented cancer cells from evading the immune surveillance, and thus exerted a consistent antitumor efficacy for heterogenic patients.^53^ Apart from single‐agent therapy, the combination of ICI agents with chemotherapy, targeted therapy, or additional ICI agents was being testified by various of clinical trials.^54^, ^55^ Recently, CheckMate 649 trial has revealed that patients with HER2‐negative GCs displayed significantly improved PFS and OS from combinational nivolumb(nivo, PD‐1 inhibitor) and chemotherapy(chemo) compared to chemotherapy alone in Western populations. This observed result was also supported by ATTRACTION 4 trial that evaluated efficacy of nivo + chemo in Asian HER2‐negative GC patients.^56^, ^57^ Both studies showed that ICI combined with chemo could be a potential first‐line treatment option for patients with GC.

It has been shown in multiple tumors, indexes such as PD‐L1 positivity, dMMR/MSI, and EBV infection are benefitable biomarkers from ICI therapy, yet TMB has not been considered valid in instructing immunotherapy in GC.^59^, ^60^, ^61^ In this study, considering patients in ICI and HER2‐targeted cohorts that shared identical HER2/*ERBB2* and TMB status and similar clinical features (comparable age and gender distribution, both in advanced stage), we speculated that immunotherapy might be a better option than HER2‐targeted therapy for advanced GC patients with positive HER2 and high TMB. Moreover, it has been shown by a series of studies that HER2 inhibition strengthened the antibody‐dependent cellular cytotoxicity while enhancing the activation of T and NK cells, potentially synergized with immune checkpoint blockade approaches.^62^ Currently, combining ICI inhibitors with HER2 inhibitors (such as Trastuzumab or Margetuximab) has been expected to display promising therapeutic efficacy against HER2‐positive breast and GC,^63^, ^64^, ^65^ and thus we inferred that the feasibility of inhibiting both HER2 and immune checkpoints in *ERBB2* ^high^TMB^high^ GC patients also deserved further investigation, whereas for *ERBB2* ^high^TMB^low^ GC patients, our data suggested that HER2‐targeted therapy remained to be a more suitable option than immunotherapy. On the other hand, the ORR, DCR, and mPFS of the two *ERBB2* ^low^ (non‐AMP/negative) groups were slightly better after received immunotherapy (33.33% [7/21], 57.14% [12/21], and 3.53 months, respectively) than received HER2‐targeted therapy (25% [3/12], 50% [6/12], and 3.05 months, respectively), suggesting that immunotherapy might also be a potential therapeutic strategy for HER2‐negative patients. Yet still, these therapeutic indications remained to be validated in larger population of GC patients.

In conclusion, our data showed that plasma‐borne *ERBB2* copy number and TMB were of great potential as progression predictors or prognostic biomarkers for HER2‐targeted in *ERBB2*‐postive GC. Further studies are warranted to validate our observation for better therapeutic management of GC patients.

## AUTHOR CONTRIBUTIONS

Cheng Zhang, Jing Gao, and Lin Shen contributed to the study conception and design. Cheng Zhang, Zuhua Chen, Xiaoyi Chong, Zhenghang Wang, and Xiaotian Zhang contributed to the data acquisition. Zuhua Chen, Xiaoxi Chen, and Yang Shao contributed to the data interpretation. Cheng Zhang, Xiaoyi Chong, Yang Chen, Ruoying Yu, and Tingting Sun contributed to the statistical analysis. All authors read and approved the final manuscript.

## CONFLICT OF INTERESTS

Ruoying Yu and Xiaoxi Chen are the employees of Geneseeq Technology Inc Canada. Tingting Sun and Yang Shao are the employee of Nanjing Geneseeq Technology Inc. The remaining authors have no conflicts of interest to declare.

## AVAILABILITY OF DATA AND MATERIALS

The datasets used in the current study are available from the corresponding authors on reasonable request.

## Supporting information

SUPPORTING INFORMATION
