# Supplementary material for: Clinical implications of plasma ctDNA features and dynamics in gastric cancer treated with HER2‐targeted therapies
Source: Clin Transl Med. 2020 Dec 15;10(8):e254. doi: 10.1002/ctm2.254 (PMC7737756; doi:10.1002/ctm2.254)
Supplement: Supplementary file 1 — SUPPORTING INFORMATION [file CTM2-10-e254-s001.docx]

**Supplementary Information**

Cheng Zhang^1†^, Zuhua Chen^2†^, Xiaoyi Chong^1†^, Yang Chen^1^, Zhenghang Wang^1^, Ruoying Yu^3^, Tingting Sun^4^, Xiaoxi Chen^3^, Yang Shao^4^, Xiaotian Zhang^1*^, Jing Gao^5*^, Lin Shen^1*^

1 Department of Gastrointestinal Oncology, Key laboratory of Carcinogenesis and Translational Research (Ministry of Education/Beijing), Peking University Cancer Hospital & Institute, 52 Fucheng Road, Beijing 100142, China.

2 Department of Oncology, Tongji Hospital, Tongji Medical College, Huazhong University of Science and Technology, Wuhan, Hubei Province, China.

3 Translational Medicine Research Institute, Geneseeq Technology Inc., Toronto, Ontario, Canada.

4 Nanjing Geneseeq Technology Inc., Nanjing, Jiangsu, 210032, China;

5 National Cancer Center/National Clinical Research Center for Cancer/Cancer Hospital & Shenzhen Hospital, Chinese Academy of Medical Sciences and Peking Union Medical College, Shenzhen, 518116, China.

† These authors contributed equally to this work: Cheng Zhang, Zuhua Chen, Xiaoyi Chong

**Correspondence**

* Professor Lin Shen, Fu-Cheng Road 52, Hai-Dian District, Beijing 100142, China. Tel: +86-10-88196561; Fax: +86-10-88196561; Email: [shenlin@bjmu.edu.cn](mailto:shenlin@bjmu.edu.cn); * Professor Jing Gao, Bao-He Road 113, Long-Gang District, Shenzhen 518000, China. Email: [gaojing_pumc@163.com](mailto:gaojing_pumc@163.com); * Professor Xiaotian Zhang, Fu-Cheng Road 52, Hai-Dian District, Beijing 100142, China. Tel: +86-10-88196561; Fax: +86-10-88196561; Email: [zhangxiaotianmed@163.com](mailto:zhangxiaotianmed@163.com);

**
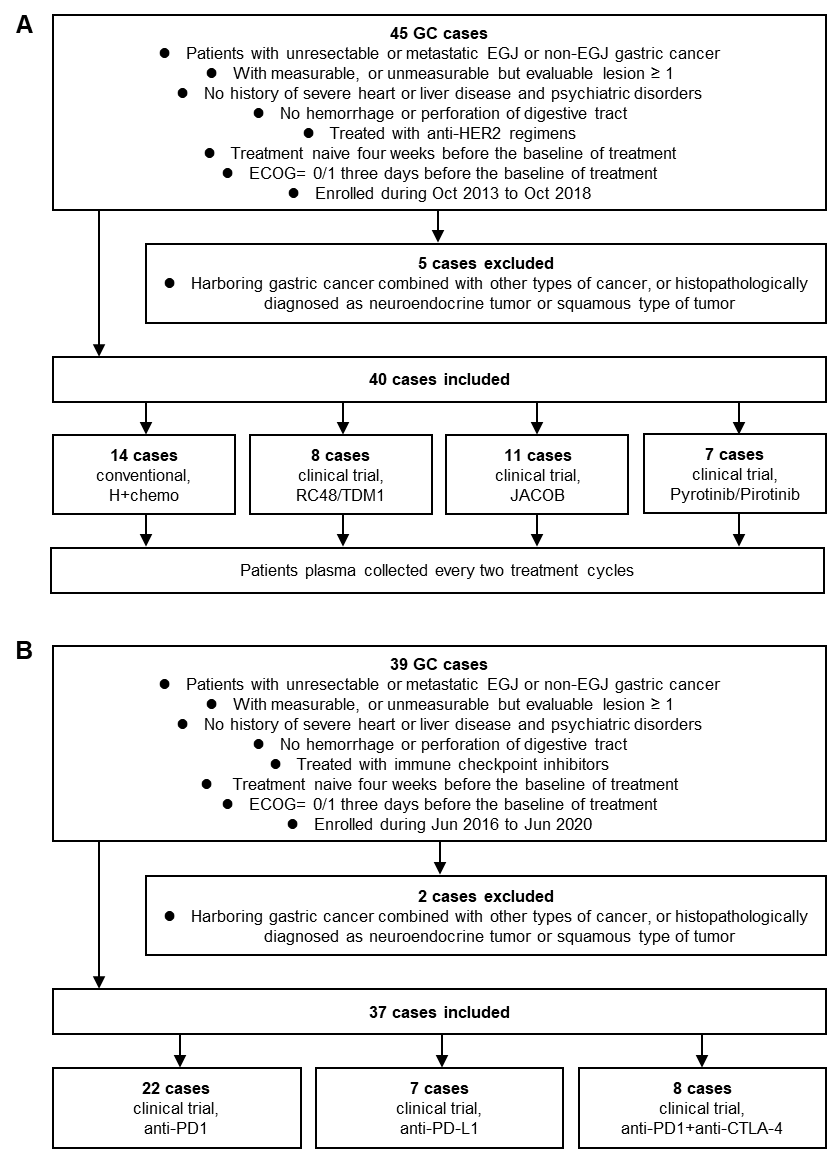
**

**Figure S1. A flow chart for the enrollment of investigated patients.** **(A)** 40 cases were enrolled into the HER2-targeted therapy cohort, while **(B)** 37 cases were enrolled in the immunotherapy cohort.

**
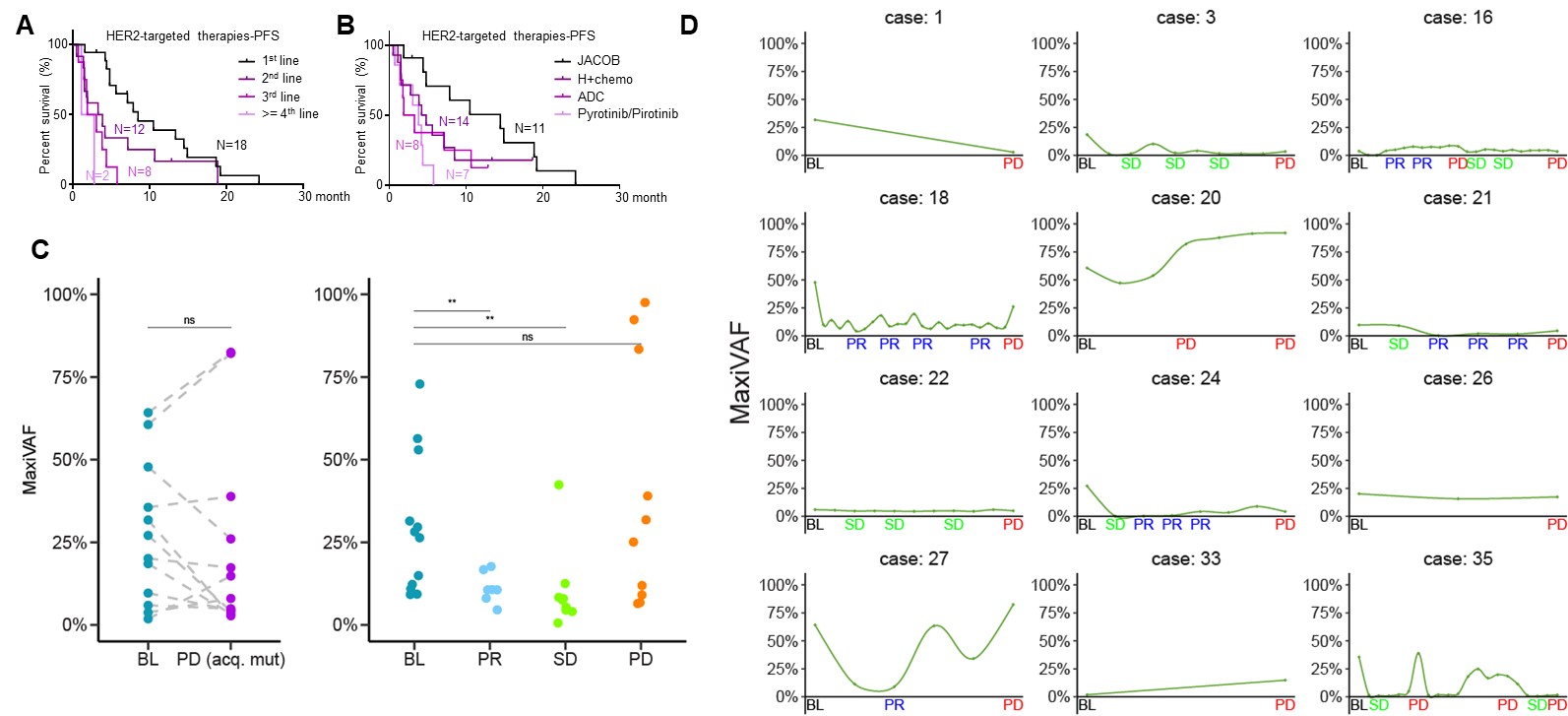
**

**Figure S2. Background features of the GC cohort received HER2-targeted therapy.** The progression-free survival proportion of the cohort was stratified by **(A)** therapeutic lines and **(B)** regimens. **(C)** The MaxiVAF of 12 patients with newly-identified mutations during HER2-targeted treatment were compared across all evaluation points. P value was performed with Wilcoxon signed rank exact test. acq.mut: acquired mutation **(D)** The real-time changes of MaxiVAF for the 12 patients with newly-identified mutations during HER2-targeted treatment.
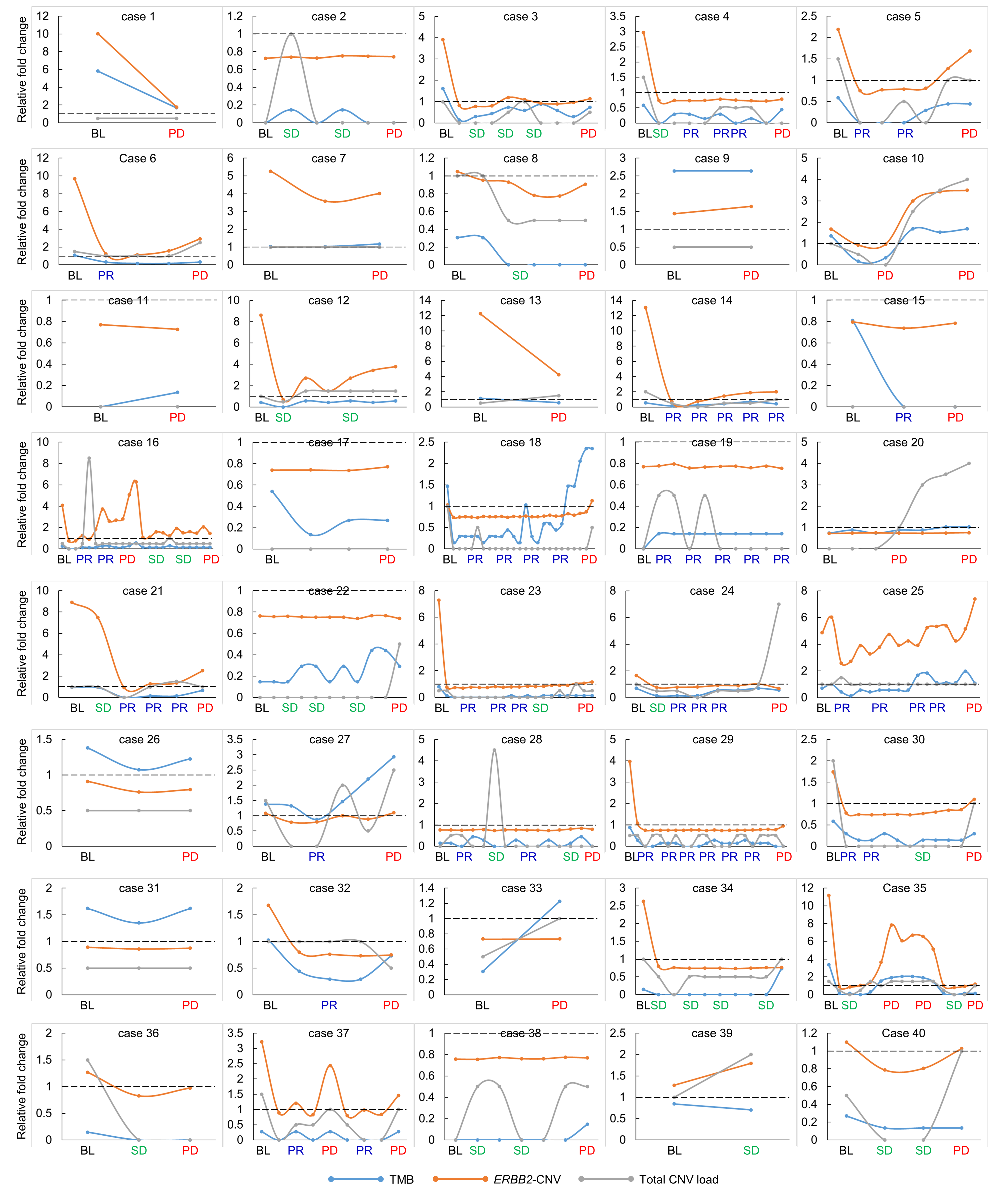


**Figure S3. The real-time changes of ctDNA genomic features (ERBB2 copy number, TCL, TMB) for all 40 patients received HER2-targeted therapy.** Levels of these 3 features were respectively normalized, and the cutoff values were formatted as a horizontal line at 1.


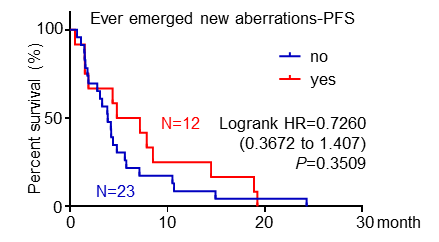


**Figure S4. Prognostic indication of emerging aberrations throughout the whole treatment.** The progression-free survival proportion of the cohort was stratified by patients with or without newly-identified aberrations including CNVs and mutations.

**Figure S5. Western blot analyses of Potential Pyrotinib-resistant mutation.** NIH-3T3 cells were transfected with wildtype/mutated forms of ERBB2 plasmids and treated with Pyrotinib, and then probed for the expression of HER2 and downstream crucial pathway members.


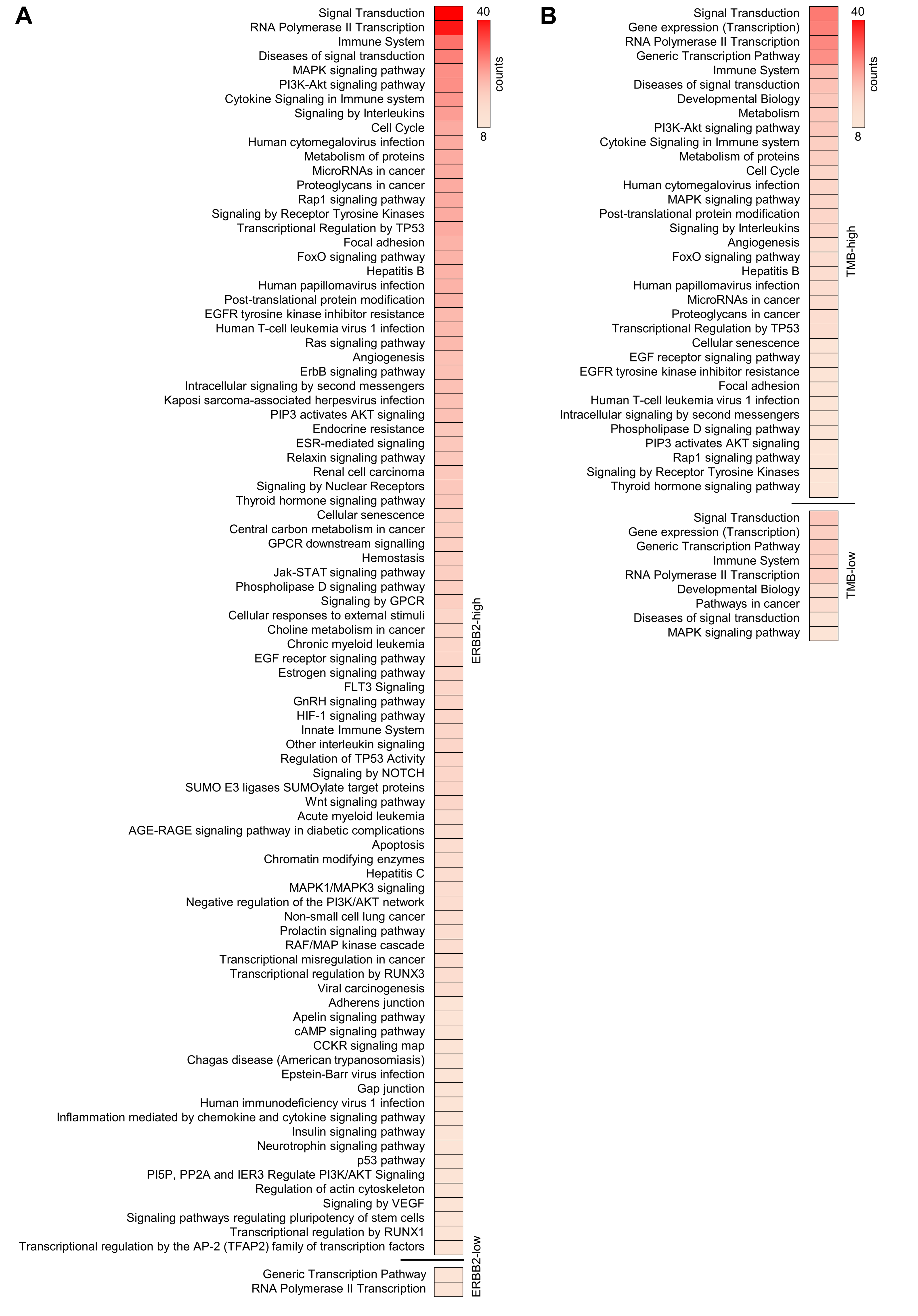


**Figure S6. Investigation of pathways enrichment for HER2-targeted patients with distinct *ERBB2* or TMB status.** For baseline samples, the CNVs and mutations identified with **(A)** *ERBB2*-classification and **(B)** *TMB*-classification were respectively analyzed for pathway enrichment.


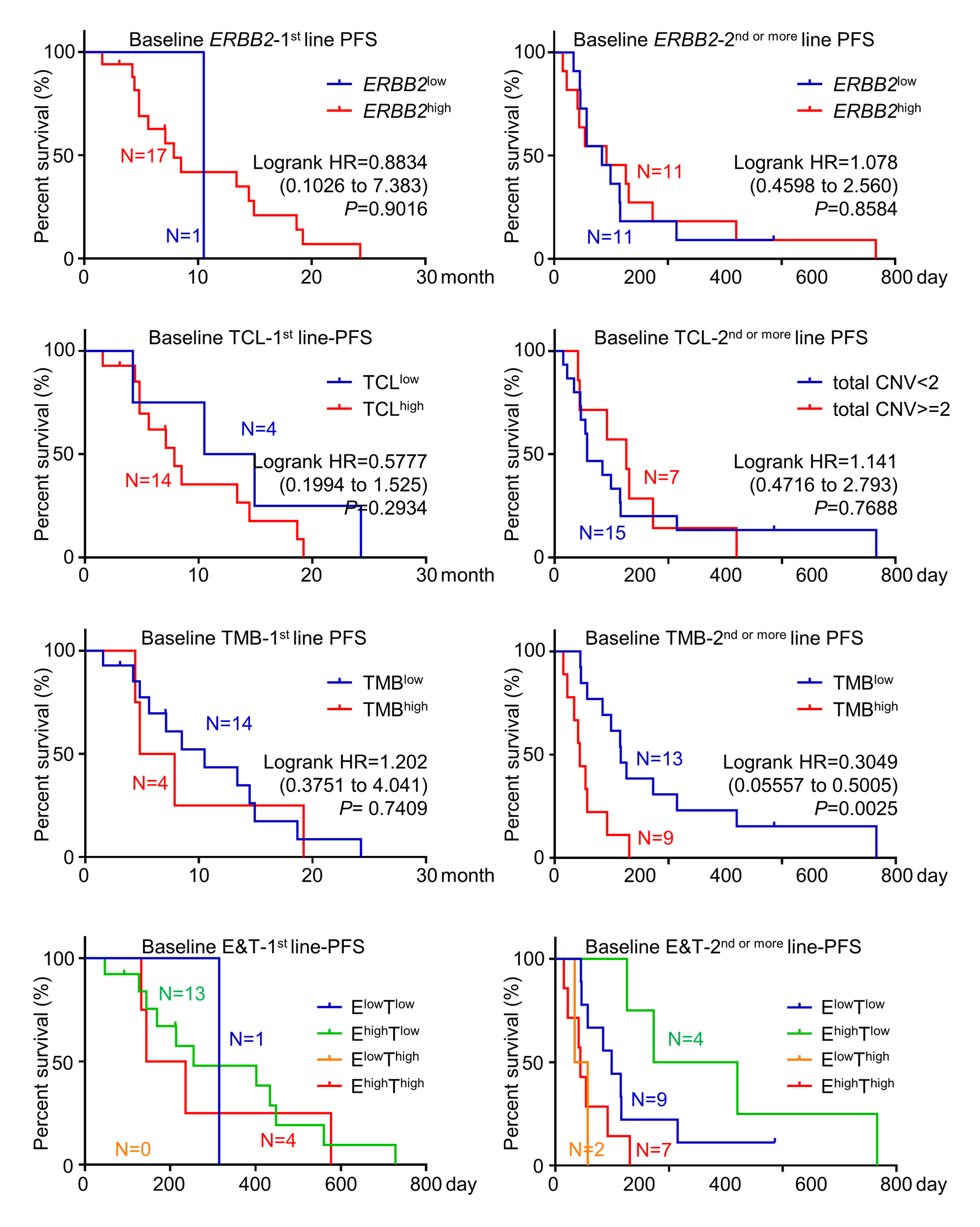


**Figure S7. The prognostic roles of genomic features for patients received different lines of HER2-targeted therapy.** PFS was analyzed for *ERBB2* copy number, TCL, TMB or *ERBB2*-TMB stratifications in patients divided into 1^st^ line and 2^nd or more^ line groups. E^low^T^low^=*ERBB2*^low^TMB^low^, E^high^T^low^=*ERBB2*^high^TMB^low^, E^low^T^high^=*ERBB2*^low^TMB^high^, E^high^T^high^=*ERBB2*^high^TMB^high^.


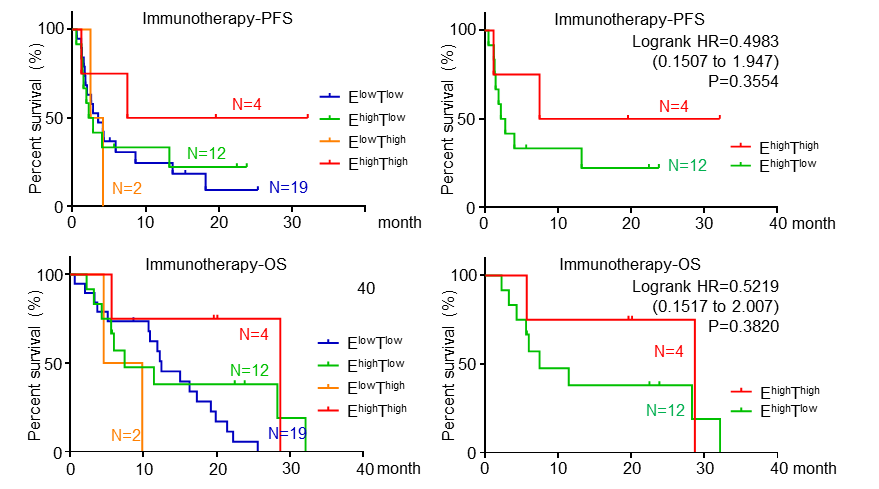


**Figure S8.** **The prognostic roles of different genomic feature for patients received immunotherapy.** PFS and OS were analyzed for ERBB2-TMB stratifications in patients. E^low^T^low^=ERBB2^low^TMB^low^, E^high^T^low^=ERBB2^high^TMB^low^, E^low^T^high^=ERBB2^low^TMB^high^, E^high^T^high^=ERBB2^high^TMB^high^.

**Table S1.** Clinicopathological characteristics of the 40 patients in HER2-targeted cohort.

| **ID** | **Sample count** | **Age** | **Gender** | **Location** | **Differentiation** | **Lauren** | **Stage** | **HER2 status** | **Regimen 1** | **Regimen 1-line** | **Regimen 2** | **Regimen-2 line** | **PFS status** | **PFS month** |
| --- | --- | --- | --- | --- | --- | --- | --- | --- | --- | --- | --- | --- | --- | --- |
| 1 | 2 | 54 | Male | non-EGJ | middle | intestinal | IV | positive | Pyrotinib | 2 | - | - | 1 | 4.2 |
| 2 | 6 | 53 | Male | non-EGJ | low | intestinal | IV | negative | JACOB | 1 | - | - | 1 | 4.8 |
| 3 | 10 | 65 | Male | non-EGJ | middle | intestinal | IV | positive | Pyrotinib | 2 | TDM1 | 3 | 1 | 1.37 |
| 4 | 10 | 57 | Female | non-EGJ | middle | intestinal | IV | positive | Pyrotinib | 3 | TDM1 | 4 | 1 | 4.37 |
| 5 | 7 | 57 | Male | non-EGJ | high | intestinal | IIIB | positive | Pyrotinib | 3 | - | - | 1 | 3.07 |
| 6 | 5 | 53 | Male | non-EGJ | low | - | IIB | positive | JACOB | 3 | - | - | 1 | 1.9 |
| 7 | 3 | 75 | Female | non-EGJ | low | diffuse | IV | positive | H+chemo | 2 | - | - | 1 | 0.5 |
| 8 | 6 | 29 | Female | non-EGJ | low | - | IV | positive | H+chemo | 2 | - | - | 1 | 1.53 |
| 9 | 2 | 49 | Male | non-EGJ | low | mixed | IIB | positive | Pyrotinib | 3 | - | - | 1 | 5.77 |
| 10 | 6 | 58 | Male | non-EGJ | low | mixed | IV | positive | Pyrotinib | 2 | - | - | 1 | 3.83 |
| 11 | 2 | 74 | Female | EGJ | low | mixed | IV | positive | JACOB | 1 | - | - | 1 | 4.4 |
| 12 | 7 | 50 | Male | non-EGJ | low | intestinal | IV | positive | H+chemo | 1 | - | - | 1 | 4.8 |
| 13 | 2 | 59 | Male | non-EGJ | middle | intestinal | IV | positive | JACOB | 1 | - | - | 1 | 7.87 |
| 14 | 6 | 60 | Male | non-EGJ | low | intestinal | IIIB | positive | H+chemo | 2 | - | - | 1 | 3.87 |
| 15 | 3 | 42 | Male | non-EGJ | middle | intestinal | IV | positive | H+chemo | 1 | - | - | 1 | 1.57 |
| 16 | 24 | 65 | Male | non-EGJ | middle | intestinal | IIIB | negative | JACOB | 2 | - | - | 1 | 10.5 |
| 17 | 4 | 67 | Male | non-EGJ | middle | intestinal | IV | positive | JACOB | 1 | - | - | 1 | 14.93 |
| 18 | 25 | 59 | Male | non-EGJ | middle | intestinal | IV | negative | H+chemo | 2 | RC48-ADC | 3 | 1 | 1.5 |
| 19 | 10 | 59 | Male | non-EGJ | low | intestinal | IV | positive | H+chemo | 1 | - | - | 1 | 7.13 |
| 20 | 7 | 56 | Female | non-EGJ | middle | intestinal | IV | positive | H+chemo | 1 | - | - | 0 | 7.07 |
| 21 | 6 | 48 | Male | non-EGJ | middle | intestinal | IV | positive | Pirotinib | 3 | - | - | 1 | 0.73 |
| 22 | 11 | 66 | Male | EGJ | low | diffuse | IV | positive | TDM1 | 2 | - | - | 1 | 7.17 |
| 23 | 21 | 63 | Female | non-EGJ | middle | - | IIIC | positive | TDM1 | 2 | - | - | 1 | 1.47 |
| 24 | 8 | 74 | Male | non-EGJ | middle | intestinal | IV | negative | JACOB | 1 | - | - | 0 | 2.97 |
| 25 | 17 | 57 | Male | EGJ | middle | intestinal | IV | positive | H+chemo | 1 | H+chemo | 2 | 1 | 5.63 |
| 26 | 3 | 54 | Male | non-EGJ | middle | intestinal | IV | positive | RC48-ADC | 2 | - | - | 1 | 1.8 |
| 27 | 6 | 72 | Male | non-EGJ | high | diffuse | IV | positive | JACOB | 1 | - | - | 1 | 14.47 |
| 28 | 15 | 60 | Male | EGJ | low | intestinal | IV | positive | H+chemo | 1 | - | - | 1 | 8.5 |
| 29 | 21 | 55 | Male | non-EGJ | low | intestinal | IV | positive | JACOB | 1 | H+chemo | 2 | 1 | 24.27 |
| 30 | 12 | 46 | Male | non-EGJ | low | mixed | IIIB | negative | RC48-ADC | 2 | - | - | 0 | 12.87 |
| 31 | 3 | 66 | Male | non-EGJ | low | intestinal | IIIB | negative | RC48-ADC | 2 | - | - | 1 | 3.3 |
| 32 | 5 | 73 | Male | EGJ | middle | intestinal | IV | positive | JACOB | 1 | - | - | 1 | 19.23 |
| 33 | 2 | 64 | Male | non-EGJ | middle | intestinal | IV | negative | RC48-ADC | 2 | - | - | 1 | 1.13 |
| 34 | 10 | 51 | Male | non-EGJ | middle | intestinal | IIIB | negative | RC48-ADC | 2 | - | - | 1 | 1.9 |
| 35 | 15 | 62 | Male | non-EGJ | low | intestinal | IV | positive | H+chemo | 1 | - | - | 1 | 4.23 |
| 36 | 3 | 69 | Female | non-EGJ | middle | intestinal | IV | positive | H+chemo | 1 | RC48-ADC | 2 | 0 | 13.4 |
| 37 | 9 | 38 | Female | non-EGJ | low | diffuse | IIIB | positive | RC48-ADC | 2 | - | - | 1 | 10.67 |
| 38 | 7 | 63 | Female | non-EGJ | middle | intestinal | IV | positive | H+chemo | 1 | - | - | 0 | 18.67 |
| 39 | 2 | 56 | Male | non-EGJ | low | diffuse | IV | negative | H+chemo | 2 | - | - | 1 | 2.8 |
| 40 | 4 | 65 | Male | EGJ | middle | intestinal | IV | positive | JACOB | 2 | RC48-ADC | 3 | 1 | 18.87 |

For PFS status, 0=progression free, 1=progressed.

For patients received two sequential lines of HER2-targeted therapy, only the PFS for regimen 1 was displayed and analyzed.

**Table S2.** List of 425 panel genes testified by NGS.

| *ABCB1* | *BRIP1* | *CYP2D6* | *FBXW7* | *IRF2* | *MRE11A* | *PIK3CA* | *RICTOR* | *TERT* |
| --- | --- | --- | --- | --- | --- | --- | --- | --- |
| *ABCB4* | *BTG2* | *CYP3A4*4* | *FGF19* | *JAK1* | *MSH2* | *PIK3R1* | *RNF43* | *TET2* |
| *ABCC2* | *BTK* | *CYP3A5* | *FGFR1* | *JAK2* | *MSH6* | *PIK3R2* | *ROS1* | *TGFBR2* |
| *ADH1A* | *BUB1B* | *DAXX* | *FGFR2* | *JAK3* | *MTHFR* | *PKHD1* | *RPTOR* | *THADA* |
| *ADH1B* | *c11orf30* | *DDR2* | *FGFR3* | *JARID2* | *MTOR* | *PLAG1* | *RRM1* | *TMEM127* |
| *ADH1C* | *CASP8* | *DENND1A* | *FGFR4* | *JUN* | *MUTYH* | *PLK1* | *RUNX1* | *TMPRSS2* |
| *AIP* | *CBL* | *DHFR* | *FH* | *KDM5A* | *MYC* | *PMS1* | *RUNX1T1* | *TNFAIP3* |
| *AKT1* | *CBLB* | *DICER1* | *FLCN* | *KDM6A* | *MYCL* | *PMS2* | *SBDS* | *TNFRSF11A* |
| *AKT2* | *CCND1* | *DLL3* | *FLT1* | *KDR* | *MYCN* | *POLD1* | *SDC4* | *TNFRSF14* |
| *AKT3* | *CCNE1* | *DNMT3A* | *FLT3* | *KEAP1* | *MYD88* | *POLD3* | *SDHA* | *TNFRSF19* |
| *ALDH2* | *CD274* | *DPYD* | *FLT4* | *KIF1B* | *MYH9* | *POLE* | *SDHB* | *TNFSF11* |
| *ALK* | *CD74* | *DUSP2* | *FOXA1* | *KIF5B* | *NAT1* | *POLH* | *SDHC* | *TOP1* |
| *AMER1* | *CDA* | *EGFR* | *FOXP1* | *KIT* | *NBN* | *POT1* | *SDHD* | *TOP2A* |
| *APC* | *CDC73* | *EML4* | *FRG1* | *KITLG* | *NCOR1* | *PPARD* | *SEPT9* | *TP53* |
| *AR* | *CDH1* | *EP300* | *GATA1* | *KLLN* | *NF1* | *PPP2R1A* | *SETBP1* | *TP63* |
| *ARAF* | *CDK10* | *EPAS1* | *GATA2* | *KMT2A* | *NF2* | *PRDM1* | *SETD2* | *TPMT* |
| *ARID1A* | *CDK12* | *EPCAM* | *GATA3* | *KMT2B* | *NFE2L2* | *PRF1* | *SF3B1* | *TSC1* |
| *ARID1B* | *CDK4* | *EPHA2* | *GATA4* | *KMT2C* | *NFKBIA* | *PRKACA* | *SGK1* | *TSC2* |
| *ARID2* | *CDK6* | *EPHA3* | *GATA6* | *KMT2D* | *NKX2-1* | *PRKACG* | *SLC34A2* | *TSHR* |
| *ARID5B* | *CDK8* | *EPHA5* | *GNA11* | *KRAS* | *NKX2-4* | *PRKAR1A* | *SLC3A2* | *TTF1* |
| *ASCL4* | *CDKN1A* | *EPHB2* | *GNAQ* | *LHCGR* | *NOTCH1* | *PRKCI* | *SLC7A8* | *TUBB3* |
| *ASXL1* | *CDKN1B* | *ERBB2* | *GNAS* | *LMO1* | *NOTCH2* | *PRKDC* | *SMAD2* | *TUBB4A* |
| *ATF1* | *CDKN1C* | *ERBB2IP* | *GRIN2A* | *LRP1B* | *NOTCH3* | *PRSS1* | *SMAD3* | *TUBB4B* |
| *ATIC* | *CDKN2A* | *ERBB3* | *GRM3* | *LYN* | *NPM1* | *PRSS3* | *SMAD4* | *TUBB6* |
| *ATM* | *CDKN2B* | *ERBB4* | *GRM8* | *LZTR1* | *NQO1* | *PTCH1* | *SMAD7* | *TYMS* |
| *ATR* | *CDKN2C* | *ERCC1* | *GSTM1* | *MAP2K1* | *NRAS* | *PTEN* | *SMARCA4* | *U2AF1* |
| *ATRX* | *CEBPA* | *ERCC2* | *GSTM4* | *MAP2K2* | *NRG1* | *PTK2* | *SMARCB1* | *UGT1A1* |
| *AURKA* | *CEP57* | *ERCC3* | *GSTM5* | *MAP2K4* | *NSD1* | *PTPN11* | *SMO* | *VAMP2* |
| *AURKB* | *CHD4* | *ERCC4* | *GSTP1* | *MAP3K1* | *NTRK1* | *PTPN13* | *SOS1* | *VEGFA* |
| *AXIN2* | *CHEK1* | *ERCC5* | *GSTT1* | *MAP3K4* | *NTRK2* | *PTPRD* | *SOX1* | *VHL* |
| *AXL* | *CHEK2* | *ESR1* | *HDAC2* | *MAP4K3* | *NTRK3* | *QKI* | *SOX14* | *WAS* |
| *B2M* | *CREBBP* | *ETV1* | *HDAC9* | *MAX* | *PAK3* | *RAC1* | *SOX2* | *WISP3* |
| *BAD* | *CRKL* | *ETV4* | *HGF* | *MCL1* | *PALB2* | *RAC3* | *SOX21* | *WRN* |
| *BAI3* | *CSF1R* | *ETV6* | *HLA-A* | *MDM2* | *PALLD* | *RAD50* | *SPOP* | *WT1* |
| *BAK1* | *CTCF* | *EWSR1* | *HNF1A* | *MDM4* | *PARK2* | *RAD51* | *SPRY4* | *XPA* |
| *BAP1* | *CTLA4* | *EXT1* | *HNF1B* | *MECOM* | *PARP1* | *RAD51B* | *SRC* | *XPC* |
| *BARD1* | *CTNNB1* | *EXT2* | *HRAS* | *MED12* | *PARP2* | *RAD51C* | *SRY* | *XRCC1* |
| *BAX* | *CUL3* | *EZH2* | *HSD3B1* | *MEF2B* | *PAX5* | *RAD51D* | *STAG2* | *YAP1* |
| *BCL2* | *CUX1* | *FANCA* | *IDH1* | *MEN1* | *PBRM1* | *RAD54L* | *STAT3* | *ZNF2* |
| *BCL2L11* | *CXCR4* | *FANCC* | *IDH2* | *MET* | *PDCD1* | *RAF1* | *STK11* | *ZNF217* |
| *BCR* | *CYLD* | *FANCD2* | *IFNG* | *MGMT* | *PDCD1LG2* | *RARA* | *STMN1* | *ZNF703* |
| *BIRC3* | *CYP19A1* | *FANCE* | *IFNGR1* | *MITF* | *PDE11A* | *RARG* | *STT3A* |  |
| *BLM* | *CYP2A13* | *FANCF* | *IGF1R* | *MLH1* | *PDGFRA* | *RASGEF1A* | *SUFU* |  |
| *BMPR1A* | *CYP2A6* | *FANCG* | *IGF2* | *MLH3* | *PDGFRB* | *RB1* | *TAP1* |  |
| *BRAF* | *CYP2A7* | *FANCI* | *IKBKE* | *MLLT1* | *PDK1* | *RECQL4* | *TAP2* |  |
| *BRCA1* | *CYP2B6*6* | *FANCL* | *IKZF1* | *MLLT3* | *PGR* | *RELN* | *TEK* |  |
| *BRCA2* | *CYP2C19*2* | *FANCM* | *IL7R* | *MLLT4* | *PHOX2B* | *RET* | *TEKT4* |  |
| *BRD4* | *CYP2C9*3* | *FAT1* | *INPP4B* | *MPL* | *PIK3C3* | *RHOA* | *TERC* |  |

**Table S3. Status of *ERBB2* in plasma and positivity of HER2/*ERBB2* in matched tissues. Paired specimens were collected at the same period before treatment (within 3 days).**

|  | **ctDNA** | |  | **Pathological** | | |  |
| --- | --- | --- | --- | --- | --- | --- | --- |
| **Case ID** | ***ERBB2*-copy number** | **diagnosis** |  | **HER2 IHC** | **FISH** | **diagnosis** | **Consistency** |
| 3 | 5.4744 | Amp |  | 3+ | - | positive | yes |
| 13 | 17.1294 | Amp |  | 3+ | - | positive | yes |
| 14 | 18.2953 | Amp |  | 3+ | - | positive | yes |
| 16 | 5.7017 | Amp |  | 3+ | - | positive | yes |
| 18 | 1.4408 | Amp |  | 2+ | Amp | positive | yes |
| 20 | 1.0112 | non-Amp |  | 2+ | non-Amp | negative | yes |
| 21 | 12.4597 | Amp |  | 3+ | - | positive | yes |
| 23 | 10.2036 | Amp |  | 3+ | - | positive | yes |
| 28 | 1.0693 | non-Amp |  | 2+ | non-Amp | negative | yes |
| 30 | 2.4377 | Amp |  | 3+ | - | positive | yes |
| 31 | 1.2498 | non-Amp |  | 1+ | - | negative | yes |
| 32 | 2.3528 | Amp |  | - | Amp | positive | yes |
| 34 | 3.6836 | Amp |  | 3+ | - | positive | yes |
| 35 | 15.6588 | Amp |  | 3+ | - | positive | yes |
| 36 | 1.7753 | Amp |  | 3+ | - | positive | yes |
| 37 | 4.4999 | Amp |  | 3+ | - | positive | yes |
| 39 | 1.7937 | Amp |  | 1+ | - | negative | no |
| 40 | 1.538 | Amp |  | 3+ | - | positive | yes |

**Table S4. The concordance between tissue (histological) HER2/*ERBB2* and plasma *ERBB2* positivity.**

|  | **ctDNA** | |  |  |
| --- | --- | --- | --- | --- |
| **Histological results** | **positive** | **negative** |  | **Total** |
| positive | 14 (93%) | 0 (0%) |  | 14 |
| negative | 1 (7%) | 3 (100%) |  | 4 |
| total | 15 | 3 |  | 18 |
| Kappa | 0.824 | |  |  |
| Concordance | 94.44%, *P*=0.000 | |  |  |

**Table S5. Genomic features in ctDNA at baseline predicted ORR and DCR to HER2-targeted therapy.**

| Baseline (BL) groups | Case number | ORR (case No.) |  | DCR (case No.) |
| --- | --- | --- | --- | --- |
| *ERBB2* | 40 |  |  |  |
| high | 28 | 60.7% (17) |  | 85.7% (24) |
| low | 12 | 25% (3) |  | 50% (6) |
| TCL | 40 |  |  |  |
| high | 21 | 61.9% (13) |  | 90.5% (19) |
| low | 19 | 36.8% (7) |  | 57.9% (11) |
| TMB | 40 |  |  |  |
| high | 13 | 38.5% (5) |  | 53.8% (7) |
| low | 27 | 55.6% (15) |  | 85.2% (23) |
| *ERBB2* and TMB |  |  |  |  |
| *ERBB2*^high^TMB^high^ | 11 | 45.5% (5) |  | 63.6% (7) |
| *ERBB2*^high^TMB^low^ | 17 | 70.6% (12) |  | 100% (17) |
| *ERBB2*^low^TMB^high^ | 2 | 0% (0) |  | 0% (0) |
| *ERBB2*^low^TMB^low^ | 10 | 30% (3) |  | 60% (6) |

**Table S6. ctDNA genomic changes at first point predicted ORR and DCR to HER2-targeted therapy.**

| Changes for stratifications (FP compared with BL) | Case number | ORR (case No.) |  | DCR (case No.) |
| --- | --- | --- | --- | --- |
| *ERBB2* | 40 |  |  |  |
| dn | 19 | 63.2% (12) |  | 94.7% (18) |
| up | 0 | - |  | - |
| remainL | 12 | 25% (3) |  | 50% (6) |
| remainH | 9 | 55.6% (5) |  | 66.7% (6) |
| TCL | 40 |  |  |  |
| dn | 14 | 64.2% (9) |  | 92.9% (13) |
| up | 3 | 0% (0) |  | 33.3% (1) |
| remainL | 16 | 43.8% (7) |  | 62.5 (10) |
| remainH | 7 | 57.1% (4) |  | 85.7% (6) |
| TMB | 40 |  |  |  |
| dn | 7 | 42.9% (3) |  | 71.4% (5) |
| up | 1 | 0% (0) |  | 0% (0) |
| remainL | 26 | 57.5% (15) |  | 88.5% (23) |
| remainH | 6 | 33.3% (2) |  | 33.3% (2) |

**Table S7. Clinicopathological characteristics of the 37 patients in immune checkpoint inhibitor-targeted cohort.**

| **ID** | **Age** | **Gender** | **Tumor location** | **Differentiation** | **Lauren** | **Stage** | **PD-L1 positivity** | **MSI status** | **Group** | **Regimen targets** | **PFS status** | **PFS month** | **OS status** | **OS month** |
| --- | --- | --- | --- | --- | --- | --- | --- | --- | --- | --- | --- | --- | --- | --- |
| 1 | 55 | Male | EGJ | low | intestinal | IV | positive | dMMR/MSI | T^low^E^high^ | PD1 | 1 | 1.93 | 1 | 5.97 |
| 2 | 57 | Male | non-EGJ | low | diffuse | IV | negative | pMMR/MSS | T^low^E^high^ | PD1 | 1 | 0.53 | 1 | 3.23 |
| 3 | 42 | Male | non-EGJ | low | mixed | IV | positive | pMMR/MSS | T^low^E^high^ | PD1 | 1 | 2.23 | 1 | 2.23 |
| 4 | 58 | Male | EGJ | middle-low | mixed | IV | positive | pMMR/MSS | T^low^E^high^ | PD1 | 1 | 2.83 | 1 | 5.6 |
| 5 | 59 | Male | non-EGJ | middle | intestinal | IV | positive | pMMR/MSS | T^low^E^high^ | PD-L1 | 1 | 1.27 | 1 | 28.3 |
| 6 | 63 | Male | non-EGJ | low | intestinal | IV | negative | pMMR/MSS | T^low^E^high^ | PD-L1 | 1 | 4.07 | 1 | 11.43 |
| 7 | 63 | Female | non-EGJ | middle | intestinal | IV | negative | pMMR/MSS | T^low^E^high^ | PD-L1 | 1 | 1.4 | 1 | 7.43 |
| 8 | 56 | Male | EGJ | middle | intestinal | IV | negative | pMMR/MSS | T^low^E^high^ | PD1 | 1 | 1.53 | 1 | 4.3 |
| 9 | 66 | Male | non-EGJ | middle-low | mixed | IV | positive | pMMR/MSS | T^low^E^high^ | PD1 | 0 | 23.83 | 0 | 23.83 |
| 10 | 76 | Male | EGJ | middle | intestinal | IV | negative | pMMR/MSS | T^low^E^high^ | PD1+CTLA-4 | 0 | 22.47 | 0 | 22.47 |
| 11 | 68 | Male | EGJ | middle-low | mixed | IV | negative | pMMR/MSS | T^low^E^high^ | PD1+CTLA-4 | 0 | 5.7 | 0 | 5.7 |
| 12 | 49 | Male | non-EGJ | low | mixed | IV | positive | - | T^low^E^high^ | PD1 | 1 | 13.27 | 1 | 32.13 |
| 13 | 60 | Female | EGJ | low | diffuse | IV | positive | dMMR/MSI | T^high^E^high^ | PD1+CTLA-4 | 1 | 1.23 | 1 | 5.67 |
| 14 | 72 | Male | non-EGJ | low | intestinal | IV | positive | dMMR/MSI | T^high^E^high^ | PD1 | 0 | 32.17 | 1 | 28.7 |
| 15 | 62 | Male | non-EGJ | middle-low | mixed | IV | positive | pMMR/MSS | T^high^E^high^ | PD1 | 1 | 7.53 | 0 | 20.1 |
| 16 | 52 | Male | non-EGJ | low | mixed | IV | negative | - | T^high^E^high^ | PD1 | 0 | 19.6 | 0 | 19.6 |
| 17 | 65 | Male | non-EGJ | low | mixed | IV | positive | dMMR/MSI | T^low^E^low^ | PD1 | 1 | 4.27 | 1 | 15 |
| 18 | 63 | Female | non-EGJ | low | mixed | IV | negative | dMMR/MSI | T^high^E^low^ | PD-L1 | 1 | 4.2 | 1 | 9.83 |
| 19 | 63 | Female | non-EGJ | low | intestinal | IV | negative | pMMR/MSS | T^low^E^low^ | PD1 | 1 | 3.53 | 1 | 10.7 |
| 20 | 56 | Female | non-EGJ | low | diffuse | IV | negative | pMMR/MSS | T^low^E^low^ | PD1 | 1 | 0.6 | 1 | 0.6 |
| 21 | 63 | Male | non-EGJ | low | diffuse | IV | negative | pMMR/MSS | T^low^E^low^ | PD1 | 1 | 2 | 1 | 2 |
| 22 | 50 | Female | non-EGJ | low | diffuse | IV | positive | pMMR/MSS | T^low^E^low^ | PD1+CTLA-4 | 1 | 2.83 | 1 | 21.43 |
| 23 | 62 | Male | EGJ | middle | intestinal | IV | - | pMMR/MSS | T^low^E^low^ | PD-L1 | 1 | 4.17 | 1 | 19.2 |
| 24 | 65 | Male | EGJ | middle | intestinal | IV | negative | pMMR/MSS | T^low^E^low^ | PD1 | 1 | 1.57 | 1 | 19.87 |
| 25 | 55 | Male | non-EGJ | low | diffuse | IV | negative | pMMR/MSS | T^low^E^low^ | PD1 | 1 | 1.8 | 1 | 10.9 |
| 26 | 60 | Male | non-EGJ | low | - | IV | negative | pMMR/MSS | T^low^E^low^ | PD1+CTLA-4 | 1 | 1.33 | 1 | 11.9 |
| 27 | 56 | Male | non-EGJ | middle | intestinal | IV | - | pMMR/MSS | T^low^E^low^ | PD1+CTLA-4 | 1 | 2.57 | 1 | 12.47 |
| 28 | 23 | Female | non-EGJ | low | diffuse | IV | positive | pMMR/MSS | T^low^E^low^ | PD1+CTLA-4 | 1 | 1.2 | 1 | 3.33 |
| 29 | 61 | Male | non-EGJ | low | diffuse | IV | positive | - | T^low^E^low^ | PD1 | 1 | 1.7 | 1 | 3.7 |
| 30 | 67 | Female | non-EGJ | middle-low | mixed | IV | positive | pMMR/MSS | T^low^E^low^ | PD1 | 1 | 5.93 | 1 | 12.23 |
| 31 | 40 | Male | non-EGJ | low | mixed | IV | positive | pMMR/MSS | T^low^E^low^ | PD1 | 1 | 13.7 | 1 | 17.3 |
| 32 | 71 | Female | EGJ | low | - | IV | positive | pMMR/MSS | T^low^E^low^ | PD1 | 0 | 25.33 | 1 | 22.27 |
| 33 | 63 | Female | EGJ | low | mixed | IV | negative | pMMR/MSS | T^low^E^low^ | PD-L1 | 0 | 15.43 | 1 | 16.3 |
| 34 | 75 | Female | non-EGJ | low | diffuse | IV | negative | pMMR/MSS | T^low^E^low^ | PD1 | 1 | 18.2 | 1 | 25.6 |
| 35 | 54 | Male | non-EGJ | low | mixed | IV | - | pMMR/MSS | T^low^E^low^ | PD1+CTLA-4 | 1 | 8.63 | 0 | 8.63 |
| 36 | 69 | Male | EGJ | middle-low | intestinal | IV | negative | - | T^low^E^low^ | PD1 | 0 | 5.13 | 1 | 5.13 |
| 37 | 60 | Male | non-EGJ | low | diffuse | IV | positive | pMMR/MSS | T^high^E^low^ | PD-L1 | 1 | 2.47 | 1 | 4.57 |

For PFS status, 0=progression free, 1=progressed; for OS, 0=living, 1=deceased.
